# Can a gender-sensitive integrated poultry value chain and nutrition intervention increase women's empowerment among the rural poor in Burkina Faso?

**DOI:** 10.1016/j.jrurstud.2023.103026

**Published:** 2023-05

**Authors:** Jessica Heckert, Elena M. Martinez, Armande Sanou, Abdoulaye Pedehombga, Rasmané Ganaba, Aulo Gelli

**Affiliations:** a Poverty, Gender, and Inclusion Unit, International Food Policy Research Institute, USA; b CGIAR Research Program on Agriculture for Nutrition and Health, International Food Policy Research Institute, USA; c Friedman School of Nutrition Science and Policy, Tufts University, USA; d Agence de Formation de Recherche et d’Expertise en Santé pour l’Afrique (AFRICSanté), USA; e Independent Consultant, Burkina Faso; f Nutrition, Diets, and Health Unit, International Food Policy Research Institute, USA

**Keywords:** Agricultural development, Burkina Faso, Gender, Mixed-methods, Poultry value chains, Women's empowerment

## Abstract

Understanding the types of food systems interventions that foster women's empowerment and the types of women that are able to benefit from different interventions is important for development policy. SELEVER was a gender- and nutrition-sensitive poultry production intervention implemented in western Burkina Faso from 2017 to 2020 that aimed to empower women. We evaluated SELEVER using a mixed-methods cluster-randomized controlled trial, which included survey data from 1763 households at baseline and endline and a sub-sample for two interim lean season surveys. We used the multidimensional project-level Women's Empowerment in Agriculture Index (pro-WEAI), which consists of 12 binary indicators, underlying count versions of 10 of these, an aggregate empowerment score (continuous) and a binary aggregate empowerment indicator, all for women and men. Women's and men's scores were compared to assess gender parity. We also assessed impacts on health and nutrition agency using the pro-WEAI health and nutrition module. We estimated program impact using analysis of covariance (ANCOVA) models and examined whether there were differential impacts by flock size or among those who participated in program activities (treatment on the treated). Program impacts on empowerment and gender parity were null, despite the program's multipronged and gender-sensitive approach. Meanwhile, results of the in-depth gender-focused qualitative work conducted near the project mid-point found there was greater awareness in the community of women's time burden and their economic contributions, but it did not seem that awareness led to increased empowerment of women. We reflect on possible explanations for the null findings. One notable explanation may be the lack of a productive asset transfer, which have previously been shown to be essential, but not sufficient, for the empowerment of women in agricultural development programs. We consider these findings in light of current debates on asset transfers. Unfortunately, null impacts on women's empowerment are not uncommon, and it is important to learn from such findings to strengthen future program design and delivery.

## Highlights

• We evaluated the impact of a gender-sensitive poultry marketing intervention on women's empowerment.
• Despite reaching and providing some benefits to women, there was no program impact on women's or men's empowerment.
• The intervention may not have targeted the areas of most significant disempowerment.
• Productive asset transfers may be essential program components for resource poor populations.

## 1 Introduction

Women around the world are often heavily involved in the labor of agricultural production and home-based processing, but they are often less involved in agricultural markets due to a range of gender-based barriers (Malapit et al., 2020; Manzanera-Ruiz et al., 2016; Njuki et al., 2022). Gender-sensitive food systems interventions that focus on increasing women's participation in value chains and agricultural markets have the potential to empower women and improve gender equality. Under some conditions, increasing women's linkages with agricultural markets has the potential to increase their control over income and intrahousehold bargaining power (David 2015; Rubin et al., 2009). However, such outcomes are not a given. First, not all women are able or desire to engage in agricultural markets. Additionally, increased participation in food systems, through processing and marketing, may not necessarily lead to increased empowerment and may depend on the structure of the local economy, gender norms, or the type of commodity (Quisumbing et al., 2021b). Poorer and more disempowered women, and those living in areas with especially restrictive gender norms, may experience more significant barriers entering markets, which raises the question of whether market-focused interventions can engage women and, if so, whether they can facilitate improved outcomes for these women and what types of programming are most conducive to women's empowerment in these circumstances.

In this study, we examine whether Soutenir l’Exploitation Familiale pour Lancer l’Elevage des Volailles et Valoriser l’Economie Rural^1^ (SELEVER), a gender-sensitive food systems intervention that focused on the poultry value chain and nutrition, impacted women's empowerment. SELEVER made different intervention components available for uptake in target villages, and participants could self-select into activities. These activities included a poultry production and marketing component, as well as a nutrition and gender component. The intervention was designed to target both production and consumption to increase the supply of and demand for poultry. SELEVER was implemented by Tanager International in western Burkina Faso and evaluated using a cluster-randomized controlled trail (cRCT), alongside formative qualitative research, a process evaluation, and in-depth qualitative research focused on gender at the midpoint of project implementation (Gelli et al., 2017). Women's empowerment is one of the pre-specified secondary trial outcomes. In this paper we examine program endline impacts on multiple dimensions of women's and men's empowerment, as well as gender parity. To preview the primary findings, we find no positive treatment effects on women's empowerment, even after considering whether there were differential impacts for those who participated in program activities (treatment on the treated analysis) or for those with larger flocks.

### 1.1 Background on women's empowerment and market-focused interventions

In this study, we draw on Kabeer's (1999) definition of empowerment as the process by which people expand their ability to make strategic life choices, particularly in contexts where this ability was previously denied to them. This definition encompasses three components: resources (access to and future claims on material human, and social resources), agency (decision-making and negotiation), and achievements (self-defined outcomes or goals). Increasingly, agricultural development interventions aim to increase women's involvement in agricultural markets and to increase their empowerment. There is a rapidly growing body of research that aims to identify the types of agricultural development interventions and implementation strategies that successfully increase women's empowerment. Most of the evidence examining what works to empower women in agriculture focuses on interventions that target women's roles in production (Quisumbing et al., 2022). Meanwhile, there have been relatively few studies to date that look at the impacts of market-focused interventions on women's empowerment, and thus, no clear evidence on whether interventions that aim to expand women's roles in either processing or marketing can help foster women's empowerment; such evidence is necessary for strengthening gender-sensitive food systems interventions (Quisumbing et al., 2021b). It is important to understand the types of interventions that successfully foster women's empowerment, as well as which women benefit from these interventions, so that the successes and failures of such programs can be used to improve such approaches.

There is some evidence that women's increased engagement in food systems, and specifically the commercialization of food systems can lead to greater empowerment of women and increased control over income (Rubin et al., 2009). A synthesis of multiple studies focused on gender and market-oriented agriculture in Asia and Africa concluded that for well-designed interventions it was possible for such projects to increase women's incomes as well as their control over their assets and incomes (Quisumbing et al., 2015). However, many of these outcomes differ by context and for populations within these contexts (David 2015; Malapit et al., 2020; Quisumbing et al., 2021b).

Additionally, lessons learned across a wide range of studies demonstrate that there are significant barriers to women's integration into markets. In a study of milk traders in peri-urban Nairobi, Kenya, Galiè and colleagues (2022) found that milk retail businesses were more profitable for men, compared to women, because of the difficulties that women experienced sourcing milk. Constraints on women's freedom of movement and their agency over how they spend their time leads them to purchase milk at higher prices and puts them at the risk of purchasing spoiled milk. Across multiple countries in sub-Saharan Africa, labor and resource (capital) constraints are the primary factors preventing women farmers from being able to produce a marketable surplus (Djurfeldt 2018). Moreover, women often lose control over assets and the sale of marketable agricultural products as products increase in value and men gain control of these products (Forsythe et al., 2016; Manzanera-Ruiz et al., 2016). In the region of Burkina Faso where SELEVER was implemented, although women frequently participate in poultry rearing, their ability to market poultry and profit from it is limited by restrictions on their interactions with male traders and physical access to places in markets where meat is sold, as well as limited access to credit and capital (Eissler et al., 2020b).

In the following sections we describe our study methods and results in detail. We then reflect on the null findings, including potential explanations for them, as well as what they mean for the design and implementation of future projects that aim to empower women through market-focused agriculture.

## 2 Description of the intervention

### 2.1 Intervention context

Burkina Faso, located in the West African Sahel, is one of the least economically developed countries and consistently ranks among the poorest 10 according to the Human Development Index (United Nations Development Programme 2021). The status of women in Burkina Faso is relatively low. Women marry early: the median age at first marriage is 17.8 years. Formal educational attainment is low, especially among women: 73.9% of women never attended school and only 22.5% are literate, compared to 59.3% and 37.6% of men, respectively (Institut National de la Statistique et de la Démographie-INSD, Burkina Faso and I.C.F. International 2012). Additionally, a relatively high share of the population, compared to other countries in the region, views wife-beating as acceptable (43.5% of women and 34.1% of men) (Institut National de la Statistique et de la Démographie-INSD, Burkina Faso and I.C.F. International 2012). Nearly half of women are anemic and 16% are underweight (BMI <18.5 kg/m2), and similarly, there are high rates of child stunting (35%), wasting (16%), anemia (88%) (Institut National de la Statistique et de la Démographie-INSD, Burkina Faso and I.C.F. International 2012).

In the Centre Ouest, Hauts-Bassins, and Boucle de Mouhoun regions where this study takes place, the two largest ethnic groups are the Mossi and Gourounsi; Peuhl, Bobo, Bwaba, and Dafing peoples are also common. Compared to the national averages, Women in Centre-Ouest have similar levels of school attendance and literacy, whereas women in Boucle du Mouhoun have slightly lower levels and women in Hauts-Bassins are slightly better off.

Burkinabe women are economically active and contribute significantly to agricultural labor. In the study areas they typically contribute labor for the farming of staple crops alongside their husbands and may be allocated an area of land for their own farming or maintain a kitchen garden near the home (Eissler et al., 2020a). Women play a key role in poultry production but have limited freedom of movement and access to poultry markets; husbands and sons typically take women's poultry to market and/or sell it to traders (Gelli et al., 2017). In the study area, there is also a sizeable gender gap in poultry ownership and access to poultry services; women own smaller flocks and have less access to veterinary and agricultural extension services (Gelli et al., 2017).

#### 2.1.1 Program design

The SELEVER intervention aimed to increase poultry production, improve the nutritional status of women and children, and empower women in the Centre Ouest, Hauts-Bassins, and Boucle de Mouhoun regions of Burkina Faso. These areas were selected because poultry production is common in these areas, and further growth of poultry production and marketing had potential given the demand in nearby urban areas. Additionally, women are commonly engaged in poultry production, as it typically occurs near the home. The program addressed poultry production, nutrition, and gender through two complementary components: one on poultry production and marketing, the other on nutrition and gender, each described in more detail below. Although gender was explicitly addressed in the second component via trainings, the focus on economic opportunities for women in the poultry component also had the potential to contribute to program impact on women's empowerment. SELEVER differed from more common nutrition-sensitive agriculture interventions in that nutrition-sensitive agriculture interventions typically emphasize sale of the surplus after household needs are met, while SELEVER aimed to cultivate marketing on a much larger scale and aimed to generate demand for poultry products beyond producer households. An intensive water, sanitation, and hygiene (WASH) component focused on health and hygiene related to poultry management was delivered to a subset of the treatment group (SELVER + WASH) (Gelli et al., 2017). Participation in each component relied on voluntary uptake, meaning that individuals could participate in the parts of the program they found appealing and participation in a component was not conditioned on participation in another.

The poultry production and marketing component included vaccinations, financing, and training on poultry flock management and housing. Of note, it did not include any sort of asset transfer, such as poultry, materials or support for a coup, vaccines, or feed. Most similar interventions in Burkina Faso do include an asset transfer. This component was implemented by two different local NGOs with similar approaches that differed in the number of members for each microfinance group. In one area, interested women and men producers were identified and organized into microfinance groups called Mutuelles de Solidarité (15–30 members each). In another area, interested women and men producers were organized into Solidarity Groups of no more than 15 people. There were multiple groups in each village, and beneficiary groups were trained by the NGO facilitators on the SELEVER poultry modules. The facilitators monitored beneficiaries’ credit use and poultry production.

Village-level vaccinators (VVVs), who are present throughout the treatment and control areas, were key community-level actors in the implementation of the SELEVER poultry component. VVVs, who had been exclusively men prior to SELEVER, are identified by the livestock services and trained on vaccination and poultry husbandry. They are trained to offer follow-up extension and vaccination services, poultry deworming and nutritional advice to the beneficiaries; these services are offered for a fee based on the number of vaccinations delivered. Additionally, government representatives from livestock extension services from the Direction régionale des ressources animales et halieutiques,^2^ provided technical support through the training and monitoring of VVVs, support to NGO facilitators for poultry trainings, market facilitation, and maintaining VVVs’ supply of vaccines and other livestock inputs. In treatment communities, VVVs received additional capacity building to address some key constraints in their ability to provide quality services. The SELEVER VVV training involved a set of targeted poultry and nutrition trainings that were provided alongside a start-up kit including a cooler and vaccines. The program also trained a cohort of female VVVs that were intended to help improve the dynamics between women producers and VVVs. By 2019, approximately 17% of the VVVs in the study area were women. VVVs were widely present and active in the full sample, but VVVs themselves are not an innovation introduced by the intervention, but the additional trainings and the availability of female VVVs were unique to the treatment areas.

The nutrition and gender curriculums had distinct content, but were fully integrated in their delivery, which was done by a different set of local NGOs from those implementing the poultry trainings. It included a behavior change communication (BCC) curriculum on nutrition and diets provided through women's groups, poultry producer groups, and local community leaders, which were delivered through trainings and home visits. The BCC activities promoted improved diets at key stages of the lifecycle, including breastfeeding, infant and young child feeding practices, and basic hygiene.

Overall, the gender component, which was based on formative research and designed by the implementing NGO's Burkinabe staff who understood both local gender norms and the types of programs being implemented globally, aimed to strengthening women's role in decision-making within households and the community on entrepreneurship, nutritious food production, marketing, consumption, and child health, feeding, and care. It also included community-level sensitization on women's economic empowerment, gender equity, and strengthening of women's groups, which included training participants from existing women's associations on enterprise development, including village savings and loans and enhancing commercial opportunities. Its delivery had four approaches. First one or two religious or traditional community leaders per village were trained on nutrition and gender themes and were responsible for training other community members with the objective that their status would contribute to the reduction in specific barriers and taboos. Additionally, champion husbands were selected based on their support for nutrition, gender, or women's poultry production. These men were trained on messaging and strategies for raising the awareness of other husbands and members of men's groups in the community. Next, two model women per village, who were identified based on their behaviors or practices, were trained on gender equity and nutrition and encouraged to identify 10 households where they would raise awareness about gender and nutrition issues and support the development of household spending plans.

Finally, women leaders were selected and trained to deliver a curriculum comprised of 10 different lessons to women's groups. After an introductory lesson, there were four lessons covering the foundation of social relationships between women and men, which included the differences between the innate and acquired differences between women and men and the foundation of inequality; the impact of behaviors, habits, and stereotypes; the roles, responsibilities, and needs of women and men; and the pressures and privileges for women and men. There were three lessons covering equity and development, which focused on understanding and imagining equity, understanding wellbeing, and identifying objectives for wellbeing in their homes, and understanding the relationship between equity and development. Finally, there were two lessons on women's roles and opportunities in poultry value chains, which covered women's representation and access to production and marketing activities in the value chain, as well as opportunities for women in the production and sale of poultry. All facilitators were first trained on dynamic facilitation techniques and the curriculum materials. The sessions were typically designed to be 30 min or 1 h long, but a few were 2 h long.

### 2.2 Summary of previous findings from the SELEVER study

Previous papers have reported on the results of the primary trial outcomes. SELEVER had only a minimal impact on diets and micronutrient intake (positive impacts on egg consumption in 2- to 4-year-olds at endline) (Becquey et al., 2022). SELEVER had a significant, although relatively small, impact on women's poultry outcomes (ownership, profits, and revenue); these impacts are primarily attributable to a shift in considering poultry as an asset that is jointly owned with male household members to labeling it women-owned (Leight et al. 2022a). Even this small shift, however, is meaningful considering that women have limited control over productive assets in this context (Njuki and Mburu 2013). With regards to a limited set of women's empowerment indicators assessed during a mid-project lean-season survey, there was no impact on either women's time use or their self-efficacy (only empowerment indicators collected in the lean season) (Leight et al., 2022b).

Notably, given SELEVER's approach, which allowed community members to self-select into activities but did not force or condition their participation, overall participation was not universal. At the mid-project lean season survey, around 57% of households in treatment communities reported any form of participation in SELEVER and 81% had been visited by a VVV; among women specifically, 37% had attended trainings related to poultry, and 29% had attended business-related trainings (Leight et al., 2022a). By endline, participation had declined somewhat. In the 12 months preceding the endline survey 24% had participated in any poultry programming activities, and 20% had participated in any nutrition-gender programming activities (Heckert et al., 2021). Although exposure may seem less than optimal for a program aiming to create change across a community, they are similar to other community-based interventions in which individuals self-select into program activities (Scott et al., 2022).

## 3 Methods

### 3.1 Trial design

SELEVER was evaluated using a cluster-randomized controlled trial in which 30 villages were assigned to each of two treatment arm (SELEVER and SELEVER + WASH) and 60 villages were assigned to the control arm. To begin 79 communes (third level administrative units) were identified as eligible based on no previous exposure to the SELEVER program pilot, year-round road accessibility, and being classified as either rural or peri-urban; 60 were randomly selected for the study. In the first stage of randomization, 30 communes each were selected for the treatment and control arms, and two villages were selected per commune, both using restricted randomization. In brief, the restricted randomization procedure modelled selection using commune- and village-level variables from the 2006 national census, including population size, existence of a government centre, number of women's associations, main agricultural crop, main source of revenue, market access, health centre presence, number of functional boreholes, and number of functional wells. An algorithm was developed using Stata to randomly allocate communes to two groups stratifying by region, and then select two villages in each commune from the list of available villages. Village selection ensured that treatment and control villages were not in close proximity, which mitigated concerns about spillover due to common VVVs and markets. The first stage of randomization forms the basis of comparisons for the combined treatment arms to the control and led to a sample of 60 communes (30 treatment and 30 control) and 120 villages (two per commune).

In the second stage of randomization, using a similar restricted randomization approach, 15 communes each were selected from the 30 treatment communes for the SELEVER and SELEVER + WASH arms. Additionally, 15 control communes were selected from the original 30 for additional trial comparisons. The second stage of randomization forms the basis for comparing the SELEVER and SELEVER + WASH arms to one another, as well as comparing each of them to the control arm and includes 45 communes (15 per arm) and 90 villages. Within each village, households were identified based on having a woman aged 15–49 years who had at least one child aged 2–4 years living in the household at baseline. Further details on the study protocol and randomization procedure have been published previously (Gelli et al., 2017).

In this paper we focus on the combined impact of the SELEVER and SELEVER + WASH treatment arms. The gender strategy did not differ between the two arms. Moreover, the women's empowerment impact results for the two arms were similar, and we also present the treatment arm specific results in the appendix. Additionally, qualitative work was integrated into the formative stage and at the study midpoint. Later in this paper, we summarize the methods and relevant findings from the midline qualitative study to triangulate findings and provide additional nuance.

### 3.2 Survey data collection

Baseline data were collected in March 2017 (post-harvest season) from 1800 households (15 households per village, 120 villages). Endline data collection began in March 2020, was postponed, due to the COVID19 pandemic, and resumed in August 2020, near the end of the agricultural slack season. Additionally, lean season surveys were conducted in September 2017 and 2019 from a subsample of 1080 households (90 villages). Each household was administered a household questionnaire focused on household characteristics and economic activities, an individual woman's survey to the woman primarily responsible for poultry activities, and an individual man's survey to the primary male decision maker. Anthropometry and other biomarker data were also collected. Attrition across the survey waves was minimal and has been described previously (Becquey et al., 2022; Leight et al. 2022a).

### 3.3 Measures of empowerment

SELEVER's impact on women's empowerment was evaluated in collaboration with the Gender Assets and Agriculture Project, Phase 2 (GAAP2). GAAP2 aimed to develop the project-level Women's Empowerment in Agriculture Index (pro-WEAI) to measure the empowerment of men and women using a portfolio of 13 agricultural development projects and to use the common metric to identify what works to empower women in these projects. Pro-WEAI is a survey-based index for measuring the empowerment, agency, and inclusion of women and men in the agriculture sector (Malapit et al., 2019). Pro-WEAI is comprised of 12 binary indicators of empowerment, a continuous aggregated empowerment score, and a binary empowerment indicator for women and men (Table 1). The 12 binary indicators of empowerment include four indicators of intrinsic agency (autonomy in income, self-efficacy, attitudes about intimate partner violence (IPV), and respect among household members); six indicators of instrumental agency (input in productive decisions, ownership of land and other assets, access to and decisions on financial services, control over use of income, work balance, and visiting important locations); and two indicators of collective agency (group membership and membership in influential groups). A respondent is considered adequate in each indicator if they have reached a certain threshold based on standard cutoffs established in Malapit et al. (2019) and described in Table 1. The continuous aggregated empowerment score is the proportion of indicators for which a respondent is adequate. A respondent is considered empowered if they achieve adequacy in at least 9 of the 12 indicators. The empowerment scores of men and women in the same household are compared to evaluate gender parity. The continuous empowerment gap indicator is the difference in empowerment scores between the man and woman in a household. Households are considered to have achieved gender parity (binary) if the woman is empowered or is at least as empowered as the man. The indicators and thresholds are based on the pro-WEAI protocols published by Malapit et al. (2019).

**Table 1:** Indicators of women's and men's empowerment.

Indicator	Level	Type	Definition
Aggregate indicators			
Empowerment score	Individual	Continuous	Proportion of indicators of empowerment respondent is adequate in
Empowered	Individual	Binary	Respondent is adequate in at least 9 of 12 indicators of empowerment
Empowerment gap	Household	Continuous	Difference between empowerment scores of man and woman in the same household
Gender parity	Household	Binary	Woman is empowered or has empowerment score at least as high as the man in the same household
Core pro-WEAI indicators			
*Intrinsic agency*			
Autonomy in income	Individual	Binary	More motivated by own values than by coercion or fear of others' disapproval; Relative Autonomy Index ≥ 1 (Ryan and EL Deci, 2000)
Self-efficacy	Individual	Binary	Agree or greater on average with self-efficacy questions in the New General Self-Efficacy Scale (Chen et al. 2001)
Attitudes about intimate partner violence	Individual	Binary	Believes husband is not justified in hitting or beating wife in all five scenarios: she goes without telling him; she neglects the children; she argues with him; she refuses to have sex with him; she burns the food
Respect among household members	Individual	Binary	Meets all of the following conditions related to the spouse, other respondent, or another household member most of the time: respondent respects relation, relation respects respondent, respondent trusts relations, respondent is comfortable disagreeing with relation
*Instrumental agency*			
Input in productive decisions	Individual	Binary	Makes decision solely, makes decision jointly and has at least some in input in the decisions, or feels could make decision for all agricultural activities they participate in
Ownership of land and other assets	Individual	Binary	Solely or jointly owns at least three assets or land
Access to and decisions on financial services	Individual	Binary	Meets one of the following conditions: belongs to a household that used a source of credit in the past year and participated in at least one decision about it; belongs to a household that did not use credit but could have if wanted to; has sole or joint access to a financial account
Control over use of income	Individual	Binary	Has input in decisions related to how to use both income and output from all agricultural activities they participate in and has in put in decisions related to income from all non-agricultural activities they participate in
Work balance	Individual	Binary	Workload less than 10.5 h per day, where workload = time spent on primary activities + (1/2)*time spend on childcare as a secondary activity
Visiting important locations	Individual	Binary	Visits at least two locations of city, market, family/relative's house at least once per week, or visits health facility or public meeting at least once per month
*Collective agency*			
Group membership	Individual	Binary	Active member of at least one community group
Membership in influential groups	Individual	Binary	Active member of at least one community group that can influence the community to at least a medium extent
Health and nutrition agency indicators			
Decides on own health and diet	Individual, women only	Binary	Has input in decisions about resting when ill, foods to prepare, and foods to eat
Decides on health and diet during pregnancy	Individual, women only	Binary	Has input in decisions about work and rest and consuming eggs, milk, and meat/poultry/fish, when pregnant
Decides on child's diet	Individual, women only	Binary	Has input in decisions about feeding eggs, milk, and meat/poultry/fish to child and feeding child when sick
Decides on weaning and breastfeeding	Individual, women only	Binary	Has input in decisions about breastfeeding, ending breastfeeding, and complementary foods
Decides to seek healthcare	Individual, women only	Binary	Has input in decisions about going to the doctor when ill and when pregnant, having another child, using contraception, taking child to doctor when sick and for well visits
Decides to purchase food and health products	Individual, women only	Binary	Has input in decisions about purchasing small and large foods, eggs, milk, meat/poultry/fish, medicines for child and self, and toiletries
Access to food and health products	Individual, women only	Binary	Able to acquire small and large foods, eggs, milk, meat/poultry/fish, medicines for child and self, and toiletries
Count versions of core pro-WEAI indicators			
New General Self-Efficacy Scale	Individual	Count	Score on the New General Self-Efficacy Scale (Chen et al., 2001)
Number of violence situations	Individual	Count	Number of scenarios in which believes husband is not justified in hitting or beating wife: she goes without telling him; she neglects the children; she argues with him; she refuses to have sex with him; she burns the food
Number of production decisions	Individual	Count	Number of agricultural activities for which makes decision solely, makes decision jointly and has at least some in input in the decisions, or feels could make decision
Number of assets owned	Individual	Count	Number of assets they own solely or jointly
Number of credit sources	Individual	Count	Number of sources of credit they participated in decisions about
Number of income decisions	Individual	Count	Number of activities for which has input in decisions related to income and outputs
Hours spent on work	Individual	Count	Number of hours spent on work
Number of visit locations	Individual	Count	Number of locations respondent visits once per week (city, market, family/relative) or once per month (health facility, public meeting)
Number of groups	Individual	Count	Number of community groups they are an active member of
Number of influential groups	Individual	Count	Number of community groups that can influence the community to at least a medium extent that they are an active member of
Poultry-specific empowerment indicators			
Decisions on poultry production	Individual	Count	Number of poultry-related activities for which makes decision solely, makes decision jointly and has at least some in input in the decisions, or feels could make decision
Owns poultry	Individual	Binary	Solely or jointly owns poultry
Decisions on poultry income	Individual	Count	Number of poultry-related activities for which has input in decisions related to income and outputs
Hours on poultry work	Individual	Count	Number of hours spent on poultry-related work

Note: Detailed indicator descriptions are available in Malapit et al. (2019) and Heckert et al. (n.d.)

Seven indicators of women's health and nutrition agency, which are part of an optional pro-WEAI add on (pro-WEAI+HN) are calculated They include decides on own health and diet, decides on health and diet during pregnancy, decides on child's diet, decides on weaning and breastfeeding, decides to seek healthcare, decides on purchasing food and health products, and access to food and health products (Heckert et al., 2023). .

Count versions of 10 of the empowerment indicators are also created to assess robustness of the findings using binary indicators. For example, the count version of the group membership indicator is the number of community groups in which a respondent is an active member. Count versions are not feasible for autonomy in income and respect among household members due to the structure of survey items used to calculate these indicators. In addition, poultry-specific empowerment indicators are constructed, including decisions on poultry production, ownership of poultry, decisions on poultry income, and hours spent on poultry-related work. Table 1 shows definitions of all empowerment indicators.

All pro-WEAI survey modules were collected at baseline and endline from the woman and man adult decision-makers in each household. Data related to work balance, self-efficacy, empowerment in nutrition and health were also collected during the two lean season surveys.

The indicators that comprise pro-WEAI were developed in collaboration with the GAAP2 portfolio which brought together project implementors and researchers focused on women's empowerment in multiple contexts. Indicator developed was also informed by complementary qualitative research (Meinzen-Dick et al., 2019). When women in SELEVER communities were asked to describe empowered women, they were often described in terms of economic empowerment, both in terms of their control over assets, their ability to make decisions about household resources, and their ability to earn money, especially in support of their families (Eissler et al., 2020b). They were also described in relation to their ability to encourage people to do things as a group and to be able to express themselves publicly. These themes map closely to many of the instrumental and collective agency indicators in pro-WEAI.

#### 3.3.1 Treatment effect estimates

Program impacts on women's and men's empowerment were estimated using analysis of covariance (ANCOVA) models.

$$Y_{ivc}=B_{0}+B_{1}S_{vc}+X_{ivc}+\varepsilon_{ivc}$$

In this model, Y is the empowerment outcome at endline for individual or household i in village v and commune c and S is whether the commune was assigned to the treatment group (either SELEVER or SELEVER + WASH). X refers to individual and/or household covariates, including household size and age of the respondent (individual-level outcomes) or age of the female respondent in the household (household-level outcomes). Standard errors are clustered at the commune level. In each table, the threshold for statistical significance is adjusted using a Bonferroni correction for the number of tests shown in that table (e.g., in a table showing six significance tests, significance is indicated where p<(0.05/6)). In addition to these primary impact estimates, we report unadjusted estimates (without covariates for household size and age) for all empowerment outcomes in Appendix Table A1.

#### 3.3.2 Robustness checks

We also conduct several robustness checks, most of which were prespecified. In line with the pre-analysis plan, we estimate treatment effects of SELEVER on the treated (those who participated in program activities) using propensity score weighted regression (Table A2). To conduct these analyses, we consider selection into the treatment group by regressing a vector of household and household characteristics at baseline (Table 3) on to program participation. We conduct this analysis using two different indicators of program participation: engagement in the nutrition and gender component and participation in SELEVER as a whole. We then estimate a difference-in-difference for households that do and do not participate.

We conduct prespecified tests for heterogeneous program impacts stratified by baseline poultry flock size. We start with the equation for estimating treatment effects described previously and add a variable for flock size>20 and an interaction term for large flock and treatment (Table A3). We also compared program impacts across the second level randomization (SELEVER treatment, SELEVER + WASH intensive treatment, and control) as prespecified. Finally, we consider potential differences that could have arisen from interrupted data collection; these could be attributable to either seasonal differences or the COVID lockdown. To do so, we start with the main impact specification and include a variable for whether data were collected before or after the lock down and an interaction term (Table A5). Notably, the COVID lockdown period in Burkina Faso was relatively short and no social safety net support was provided during this period.

## 4 Results

Nearly all households in the sample were dual-adult households, defined as having at least one male and one female adult. Households were large (average 8.8 household members) and on average had more children under 15 than adults, and about half of households were polygynous. The male respondent was about 10 years older than the female respondent on average. Most men and women respondents did not speak French and were not literate in the local language (Table 2).

**Table 2:** Household and individual characteristics at baseline.

	Control		Treatment		p-value
	Mean	n	Mean	n	
Household size	8.69	890	8.71	873	0.923
Dual-adult household	0.97	890	0.98	873	0.281
Dependency ratio	1.35	890	1.34	873	0.519
Number of children under 15	4.66	890	4.61	873	0.713
Age of woman	33.31	885	33.34	871	0.954
Age of man	43.70	777	44.06	774	0.570
Woman is married	0.98	883	0.97	871	0.184
Man is married	0.98	777	0.97	774	0.319
Woman speaks French	0.09	883	0.09	871	0.943
Man speaks French	0.25	777	0.22	774	0.200
Woman is literate in local language	0.05	883	0.08	871	0.009*
Man is literate in local language	0.13	777	0.12	774	0.456
Polygynous	0.46	885	0.47	871	0.683
Woman was pregnant in last two years	0.47	884	0.45	871	0.472
Woman has child under 2	0.36	884	0.35	871	0.751

*p < 0.05.

**Table 3:** Women's and men's empowerment at baseline.

	Control		Treatment		p-value
	Mean	n	Mean	n	
**Aggregate empowerment indicators**					
Empowerment score (woman)	0.52	754	0.51	721	0.105
Empowerment score (man)	0.68	634	0.69	657	0.090
Empowered (woman)	0.12	754	0.09	721	0.029*
Empowered (man)	0.43	634	0.47	657	0.187
Empowerment gap (household)	0.17	610	0.18	627	0.113
Gender parity (household)	0.28	610	0.24	627	0.099
**Core pro-WEAI indicators (binary)**					
Autonomy in income (woman)	0.58	885	0.57	871	0.631
Autonomy in income (man)	0.68	777	0.68	773	0.798
Self-efficacy (woman)	0.45	885	0.43	871	0.273
Self-efficacy (man)	0.59	777	0.63	773	0.092
Attitudes about IPV (woman)	0.52	885	0.55	871	0.222
Attitudes about IPV (man)	0.66	777	0.71	773	0.038*
Respect among household members (woman)	0.64	854	0.68	844	0.118
Respect among household members (man)	0.71	732	0.71	767	0.966
Input in productive decisions (woman)	0.79	885	0.80	871	0.471
Input in productive decisions (man)	0.96	777	0.95	773	0.109
Ownership of land and other assets (woman)	0.83	885	0.84	871	0.346
Ownership of land and other assets (man)	0.99	777	0.99	773	0.443
Access to and decisions on financial services (woman)	0.20	885	0.21	871	0.866
Access to and decisions on financial services (man)	0.38	777	0.45	773	0.007*
Control over use of income (woman)	0.67	885	0.67	871	0.905
Control over use of income (man)	0.87	777	0.87	773	0.850
Work balance (woman)	0.31	782	0.31	746	0.865
Work balance (man)	0.76	679	0.74	663	0.313
Visiting important locations (woman)	0.51	885	0.55	871	0.175
Visiting important locations (man)	0.73	777	0.74	773	0.688
Group membership (woman)	0.40	885	0.29	871	<0.001*
Group membership (man)	0.44	777	0.43	773	0.565
Membership in influential groups (woman)	0.30	885	0.24	871	0.007*
Membership in influential groups (man)	0.37	777	0.40	773	0.333
**Core pro-WEAI indicators (count)**					
New General Self-Efficacy score (woman)	28.20	885	28.05	871	0.589
New General Self-Efficacy score (man)	29.67	777	30.43	773	0.008*
Number of violence situations (woman)	3.61	885	3.54	871	0.372
Number of violence situations (man)	4.17	777	4.25	773	0.238
Number of production decisions (woman)	6.66	859	6.74	845	0.780
Number of production decisions (man)	15.06	767	14.49	758	0.100
Number of assets owned (woman)	3.49	885	3.64	871	0.120
Number of assets owned (man)	6.87	777	6.98	773	0.309
Number of credit sources (woman)	0.22	885	0.22	871	0.890
Number of credit sources (man)	0.48	777	0.56	773	0.024*
Number of income decisions (woman)	6.25	885	6.41	871	0.583
Number of income decisions (man)	14.82	777	14.21	773	0.087
Hours spent on work (woman)	6.43	782	6.93	746	0.006*
Hours spent on work (man)	4.77	679	5.26	663	0.037*
Number of visit locations (woman)	1.51	885	1.56	871	0.332
Number of visit locations (man)	2.24	777	2.28	774	0.566
Number of groups (woman)	0.55	885	0.39	871	<0.001*
Number of groups (man)	0.65	777	0.59	773	0.163
Number of influential groups (woman)	0.42	885	0.32	871	0.003*
Number of influential groups (man)	0.54	777	0.54	773	0.996
**Poultry-specific empowerment indicators**					
Decisions on poultry production (woman)	2.70	536	2.92	524	0.106
Decisions on poultry production (man)	5.35	710	5.16	695	0.005*
Owns poultry (woman)	0.24	885	0.24	871	0.641
Owns poultry (man)	0.90	777	0.90	773	0.839
Decisions on poultry income (woman)	2.57	536	2.84	524	0.057
Decisions on poultry income (man)	5.31	710	5.11	695	0.006*
Hours on poultry work (woman)	0.04	782	0.02	746	0.396
Hours on poultry work (man)	0.38	679	0.33	663	0.412
**Indicators of nutrition and health agency**					
Decides on own health and diet (woman)	0.62	884	0.54	871	<0.001*
Decides on health and diet during pregnancy (woman)	0.72	414	0.78	393	0.077
Decides on child's diet (woman)	0.81	884	0.80	871	0.526
Decides on weaning and breastfeeding (woman)	0.97	318	0.95	307	0.483
Decides on seeking healthcare (woman)	0.85	764	0.85	742	0.755
Decides on purchasing food and health products (woman)	0.06	890	0.05	873	0.092
Access to food and health products (woman)	0.31	890	0.37	873	<0.001*
*p < 0.05					

Empowerment of both women and men was low at baseline. Woman achieved adequacy in about half of the 12 indicators of empowerment, and about 10 percent of women were empowered; men achieved adequacy in about two-thirds of the 12 indicators of empowerment, and just under half of men were empowered. Few households achieved gender parity, meaning that women were rarely empowered or as empowered as the man in their household. Across all 12 indicators of empowerment, a higher proportion of men than woman achieved adequacy. Most men and women had adequate input in productive decisions, ownership of land and other assets, respect among household members, and control over use of income. Most men and women did not have adequate access to and control over financial services, group membership, and membership in influential groups. The gap between men and women was largest for work balance, where about 30 percent of women experienced work balance (meaning that they were not overworked), while about 75 percent of men experienced work balance (Table 3).

**Fig. 1:**
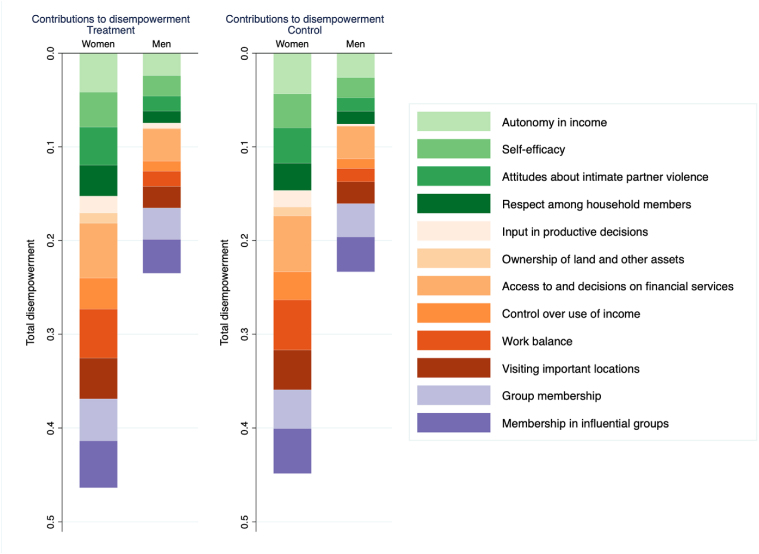
Contributors to disempowerment by treatment group Note: The figure compares the depth of disempowerment among women and men in the treatment and control groups; longer bars indicate higher levels of disempowerment. It also depicts the breakdown of the largest contributors to disempowerment among these same groups; the contribution of each indicator is depicted by a different color. (For interpretation of the references to color in this figure legend, the reader is referred to the Web version of this article.)

The pro-WEAI data were also used to assess how much each indicator of empowerment contributed to women's and men's disempowerment. For both women and men, the largest contributors to disempowerment in the study population were access to and decisions on financial services, work balance, and membership in influential groups (Fig. 1).

Descriptive analyses revealed few changes in women's and men's empowerment between baseline and endline. There were small increases in the percentage of women and men adequate in self-efficacy (1.09 and 0.95 percentage points (pp) increase, respectively), a small decrease in the percentage of men adequate in autonomy in income (−1.54 pp), and a small increase in the percentage of households that achieved gender parity (3.34 pp).

In terms of treatment effects, ANCOVA models revealed that there was no significant impact of the SELEVER program at endline on women's or men's empowerment when looking at the aggregate empowerment score or the binary empowered variable (Table 4). Additionally, there were no significant impacts on gender parity or the empowerment gap between women and men in the same household.

**Table 4:** Treatment effect estimates: aggregate empowerment indicators.

	(1)	(2)	(3)	(4)	(5)	(6)
	Empowerment score (women)	Empowerment score (men)	Empowered (women)	Empowered (men)	Empowerment gap	Gender parity
Treatment	−0.01	−0.00	0.00	−0.00	0.01	−0.00
	(0.01)	(0.01)	(0.02)	(0.04)	(0.01)	(0.03)
Observations	1487	1396	1487	1396	1327	1327
R-squared	0.011	0.006	0.003	0.007	0.012	0.007

Standard errors clustered at the commune level are shown in parentheses.

In Columns 1–4 treatment estimates control for household size and age of respondent.

In Columns 5–6 treatment estimates control for household size and age of female respondent in the household.

*p < 0.0083 (threshold adjusted using Bonferroni correction for 6 tests).

The aggregate pro-WEAI indicators provide an overall view of empowerment, but they may mask changes in different directions for the individual indicators. Thus, in the next part of the analysis, we look more carefully at the individual indicators (both binary and continuous), as well as the aspects of these indicators that specifically pertain to poultry rearing. After adjusting for multiple comparisons, there were no significant impacts on women's or men's empowerment when looking at the individual binary indicators that comprise pro-WEAI (Table 5) or indicators of empowerment in nutrition and health (Table 6), count indicators of empowerment (Table 7), or poultry-related indicators of empowerment (Table 8). (Prior to applying Bonferroni corrections, the only significant impact was a decrease in women's perception of mutual respect among household members.) Of note, there is reason for the null finding related to women's work balance to be interpreted in a positive light. The SELEVER intervention included multiple activities that could have increased women's time burden, and the program also focused on raising awareness of women's time burden among men and community members. Thus, the lack of negative impact on work balance may be the result of programmatic efforts to mitigate the intervention's burden on women's time.

**Table 5:** Treatment effect estimates: core pro-WEAI indicators for women and men.

	(1)	(2)	(3)	(4)	(5)	(6)	(7)	(8)	(9)	(10)	(11)	(12)
	Autonomy in income	Self-efficacy	Attitudes about IPV	Respect among household members	Input in productive decisions	Ownership of land and other assets	Access to and decisions on financial services	Control over use of income	Work balance	Visiting important locations	Group membership	Membership in influential groups
**Panel A: Women**												
Treatment	0.05	−0.01	−0.03	−0.05	0.01	−0.01	0.03	−0.03	0.01	−0.01	−0.04	−0.02
	(0.03)	(0.03)	(0.04)	(0.03)	(0.03)	(0.02)	(0.02)	(0.04)	(0.03)	(0.03)	(0.03)	(0.03)
Observations	1624	1624	1624	1487	1624	1624	1624	1624	1624	1624	1624	1624
R-squared	0.003	0.008	0.007	0.008	0.006	0.001	0.004	0.007	0.029	0.001	0.006	0.006
**Panel B: Men**												
Treatment	0.01	−0.03	−0.04	−0.01	−0.03	−0.01	0.03	0.00	−0.03	−0.02	0.05	0.04
	(0.03)	(0.03)	(0.02)	(0.03)	(0.01)	(0.01)	(0.04)	(0.02)	(0.03)	(0.03)	(0.04)	(0.04)
Observations	1472	1472	1472	1400	1472	1472	1472	1472	1467	1472	1472	1472
R-squared	0.000	0.067	0.003	0.002	0.009	0.008	0.012	0.006	0.025	0.008	0.009	0.007

Standard errors clustered at the commune level are shown in parentheses.

Treatment estimates control for household size and age of respondent.

*p < 0.0042 (threshold adjusted using Bonferroni correction for 12 tests).

**Table 6:** Treatment effect estimates: indicators of nutrition and health agency, women.

	(1)	(2)	(3)	(4)	(5)	(6)	(7)
Respondent decides on …	Own health and diet	Health and diet during pregnancy	Child's diet	Weaning and breastfeeding	Seeking healthcare	Purchasing food and health products	Access to food and health products
Treatment	−0.00	−0.04	−0.01	−0.00	0.02	0.01	0.02
	(0.03)	(0.06)	(0.03)	(0.03)	(0.02)	(0.03)	(0.03)
Observations	1624	254	1624	553	1125	1626	1626
R-squared	0.000	0.007	0.001	0.000	0.014	0.004	0.002

Standard errors clustered at the commune level are shown in parentheses.

Treatment estimates control for household size and age of respondent.

*p < 0.0071 (threshold adjusted using Bonferroni correction for 7 tests).

**Table 7:** Treatment effect estimates: count versions of core pro-WEAI indicators.

	(1)	(2)	(3)	(4)	(5)	(6)	(7)	(8)	(9)	(10)
	New General Self-Efficacy score	Number of violence situations	Number of production decisions	Number of assets owned	Number of credit sources	Number of income decisions	Hours spent on work	Number of visit locations	Number of groups	Number of influential groups
**Panel A: Women**										
Treatment	−0.30	−0.07	−0.56	−0.04	0.03	−0.43	−0.05	0.05	−0.03	−0.01
	(0.32)	(0.12)	(0.47)	(0.13)	(0.03)	(0.43)	(0.35)	(0.06)	(0.05)	(0.04)
Observations	1624	1624	1574	1624	1624	1624	1624	1626	1624	1624
R-squared	0.014	0.006	0.007	0.007	0.003	0.007	0.010	0.002	0.006	0.006
**Panel B: Men**										
Treatment	0.03	−0.04	−0.57	−0.01	0.09	−0.38	0.00	0.05	0.11	0.11
	(0.27)	(0.06)	(0.41)	(0.21)	(0.07)	(0.36)	(0.41)	(0.07)	(0.06)	(0.06)
Observations	1472	1472	1462	1472	1472	1472	1467	1472	1472	1472
R-squared	0.093	0.002	0.025	0.011	0.013	0.024	0.037	0.013	0.008	0.008

Standard errors clustered at the commune level are shown in parentheses.

Treatment estimates control for household size and age of respondent.

*p < 0.005 (threshold adjusted using Bonferroni correction for 10 tests).

**Table 8:** Treatment effect estimates: Poultry-specific empowerment indicators.

	(1)	(2)	(3)	(4)
	Decisions on poultry production	Owns poultry	Decisions on poultry income	Hours on poultry work
**Panel A: Women**				
Treatment	0.07	0.09	0.22	0.00
	(0.24)	(0.05)	(0.25)	(0.00)
Observations	1093	1624	1093	1624
R-squared	0.013	0.018	0.011	0.001
**Panel B: Men**				
Treatment	−0.02	−0.02	0.02	0.02
	(0.06)	(0.02)	(0.06)	(0.03)
Observations	1362	1472	1362	1467
R-squared	0.002	0.015	0.003	0.006

Standard errors clustered at the commune level are shown in parentheses.

Treatment estimates control for household size and age of respondent.

*p < 0.0125 (threshold adjusted using Bonferroni correction for 4 tests).

### 4.1 Robustness checks

In conducting multiple robustness checks, we did not find any evidence to support a revised interpretation to our main quantitative finding that the SELEVER program did not empower women or men or increase gender parity. The two separate estimates of treatment on the treated took into account the propensity to participate in the nutrition and gender component, specifically, as well as the overall program. The only positive impact, for women, after accounting for multiple hypothesis testing, was an increase in membership in influential groups among women who participated in SELEVER as a whole (Appendix Table A2). This finding is logical given that the exposure variable includes participation in SELEVER-related groups. Additionally, men in households that participated in the nutrition and gender component were more likely to be members of a community group. After accounting for multiple hypothesis testing, there were no additional impacts detected.

We examined potential heterogenous impact for those who owned large (>20) compared to small flocks at baseline. After accounting for multiple hypothesis testing, we did not find significant differences in impacts on any of the empowerment outcomes among those with large flocks (Appendix Table A3).

We considered the potential for differential impacts of the SELEVER and SELEVER + WASH treatment arms. After accounting for multiple hypothesis testing, we did not find a significant impact on any of the empowerment outcomes for either of the treatment arms (Appendix Table A4). We did identify a significant difference between the two arms on whether women decide on their own health and diet during pregnancy; one was positive, the other negative, and neither significantly different from zero.

We considered that the impact of the treatment may have differed according to whether the endline data were collected before or after the COVID lockdown, with data collection occurring near the end of the agricultural slack season. We found limited support for this hypothesis (Appendix Table A5). In looking at the continuous version of each indicator, we find that women in the treatment arm who were surveyed in August (following the lockdown) spent slightly less time working. In this case it is difficult to disentangle the effects of the lockdowns from the effects of the agricultural slack season that was underway in August. Daily life during COVID in rural Burkina Faso went on as normal, and there was no pandemic-related government support. Additionally, it is worth noting that previous work also found that SELEVER had no impact on women's time use in the lean season (Leight et al., 2022a).

### 4.2 Summary of qualitative methods and findings

The SELEVER study also conducted in-depth qualitative gender research near the midpoint of the SELEVER intervention. The findings from these studies have been reported elsewhere (Eissler et al., 2020a, 2020b). In this section we summarize the methods and key results from the qualitative work that can be triangulated with the quantitative findings presented above.

Qualitative data were collected in six purposefully selected SELEVER villages across five provinces. Data collection in each village included four sex-disaggregate focus groups (two on gender norms, and two on nutrition and food production and allocation) and semi-structured interviews were conducted with a man and a woman from two different households. Additionally seasonal calendars were conducted in half the villages and key-informant interviews were conducted with 13 group leaders, 10 VVVs, and six poultry traders. The discussion guides for the focus groups and interviews were heavily informed by the pro-WEAI qualitative protocols (Meinzen-Dick et al., 2019), and the nutrition and food production and allocation focus group was informed by vignettes developed by Elias et al. (2018).

Overall, the qualitative work concludes that at the midpoint of the program there were some small changes related to women's empowerment and gender norms that participants attributed to the program. Firstly, men often acknowledged, and attributed to SELEVER, their awareness of the common division of labor that burdens women's time, the value of discussing important decisions with their wives, and the importance of women's economic contributions to the household (Eissler et al., 2020a). This finding is in line with the interpretation that the null effect on work balance can be interpreted as the project's successful mitigation of women's increased work due to the intervention.

Additionally, participants described increased acceptance of women in public places, such as markets, which has coincided with the SELEVER program, but that community members did not specifically attribute to the program (Eissler et al., 2020a). Part of the shift in accepting women in public spaces may be attributable to the cadre of women VVVs that were trained by SELEVER (Eissler et al., 2020a, 2020b). Given the lack of impact on the frequency of women visiting important places, it seems that these changes in men's perceptions and the behaviors of some women in the community did not trickle down to actual changes in household gender relationships during the period covered by the study. Overall, evidence from the qualitative study concludes that although SELEVER has led to some gender-related changes, they may be relatively small-scale changes in terms of moving the needle on women's empowerment.

## 5 Discussion

Our results provide rigorous evidence that SELEVER, a multisectoral gender- and nutrition-sensitive intervention that focused on the poultry value chain in western Burkina Faso, did not lead to measurable increases in women's empowerment across program beneficiaries. An exception to this is that the null finding on women's work balance suggests that the intervention may have been able to mitigate women's increased labor burden due to the intervention. The lack of impact on women's empowerment occurs even though the program implemented a range of gender-sensitive strategies (Johnson et al., 2018) and the study measured women's empowerment using the pro-WEAI, which is sensitive enough to detect impact in similar types of projects with comparable or smaller sample sizes (Quisumbing et al., 2022; Seymour et al.). Further analysis, including the effects of treatment on the treated and comparing impacts by flock size, similarly found no impact on women's empowerment. These results leave unanswered questions as to why there were no detectable impacts on women's empowerment. In this discussion we consider potential explanations for the null findings. We elaborate these explanations with supporting qualitative evidence from SELEVER, recent findings from other gender-sensitive agricultural development projects, and the results of the primary trial outcomes published elsewhere (Becquey et al., 2022; Leight et al. 2022a).

### 5.1 Explanations for null results

We consider three potential explanations for SELEVER's lack of impact on women's empowerment. First, SELEVER may have used too light of a touch in its intervention, a hypothesis that has also been considered as an explanation for the limited impact on poultry production (Leight et al., 2022a). Second, there are high levels of disempowerment in the study population, and SELEVER may not have targeted the domains of empowerment where women are most disempowered. Finally, the study population may have been too poor to fully invest in the demands of the market-oriented intervention. Yet, despite the high levels of poverty in the study area, SELEVER did not transfer assets, further limiting the potential for impact. We explore each of these explanations in greater depth.

The first explanation for the lack of program impact is that SELEVER may have been too light in terms of the depth of activities delivered, as well as participation by intended beneficiaries. SELEVER primarily delivered information to producers and consumers to strengthen market linkages and chose to focus on providing a lighter intervention with small amounts of exposure to these services to a larger number of beneficiaries, instead of targeting fewer beneficiaries more intensely. The results of SELEVER's impact on poultry production outcomes demonstrate that about half of households participated in some capacity in earlier stages of the intervention, but engagement faded with time, and that there were only small impacts on poultry production outcomes (Leight et al., 2022a). Gender trainings were delivered along with the nutrition component, but the gender focus also cut across other aspects of the design, especially in terms of the gender-sensitive nature of the project's approach to increasing women's involvement in and empowerment from poultry production. However, the lack of integrated coverage of program components may have limited the potential for program impacts on women's empowerment.

Another explanation is that, although SELEVER prepared a multipronged, comprehensive, and culturally appropriate gender strategy that used multiple entry points in the community (i.e., leaders, men, producer groups, peer support, and women's group) to increase women's empowerment, it may not have targeted the areas of empowerment where women were most disempowered and that could have had the biggest influence on women's lives. In decomposing the contributors to disempowerment using the pro-WEAI, we found that the three largest areas of disempowerment for women in the study area at baseline were “access to and decisions on credit and financial services,” “work balance,” and “membership in influential groups.“^3^ Although SELEVER aimed to link women with existing credit sources, this aspect of the program was not particularly successful, as no impact was found on women taking loans (Leight et al., 2022a). Qualitative findings suggest that the size and terms of the loans available via SELEVER partners were often not agreeable to women who sought these loans, often because payments were due at times that did not coincide with when women made profits from poultry rearing (Eissler et al., 2020b). Additionally, women must often seek their husband's permission to participate in savings and loans groups, and men often request that their wives take loans for men's own use (Eissler et al., 2020b).

Additionally, the potential of a market-focused intervention like SELEVER to empower women relies a great deal on women's ability to maintain control over the income they are generating. The qualitative study results found that it may have been difficult for women to maintain strong control over poultry-related income. Women in SELEVER communities indicated that the most common source of disagreement with their spouses was how to manage and invest income, especially poultry income (Eissler et al., 2020a). Regardless of who is involved in poultry rearing activities, it is considered the male household head's decision whether to slaughter or kill a chicken, and a woman would not be able to do so without her husband's permission, even if she owned the chicken (Eissler et al., 2020b). It is commonly expected in the study area that women would receive the income from any of her poultry sold by her husband and that she can allocate it to specific needs in the household. In practice, however, she may have limited overt control over this income, especially when her husband knows the amount and when she received it.

A final explanation for the lack of impact on women's empowerment may be that the depth of both disempowerment and poverty in Burkina Faso are too much to overcome in the scope of a community-level development project that functions over a relatively short period and at relatively low intensity, especially when that intervention does not provide asset transfers. As described earlier in this paper, Burkina Faso consistently ranks among the poorest10 countries according to the Human Development Index and that women's educational attainment and literacy are low, while the acceptability of wife beating is high. It is worth noting that interventions that take place in countries with similarly low levels of human development and poor conditions for women have demonstrated impacts on women's empowerment using pro-WEAI (Benali et al., 2020; Hillesland et al., 2022; Janzen et al., 2018; Quisumbing et al., 2021a), as have other interventions implemented in Burkina Faso that measured women's empowerment with other approaches (Heckert et al. 2019; Olney et al., 2016).

Amidst such conservative gender contexts, it is also possible that interventions that aim to empower women and transform gender norms might be deemed as too progressive and lead to backlash from men or cause women to disengage to avoid backlash (Baland and Guirkinger 2022). The qualitative part of the study found that men were generally supportive of their wives' income generating activities and that the project increased their openness to their wives' input into decisions (Eissler et al., 2020a). At the same time, there were conflicts when these activities prevented women from fulfilling other household duties (e.g., cooking, childcare) and that women often had to negotiate to work around these competing demands. It may have been difficult for women to gain support for activities with less obvious benefits, such as attending a meeting. Additionally, one of the pro-WEAI indicators, acceptability of IPV, was included in the index to be able to consider potential backlash in the form of IPV, and there were no program-related impacts on this indicator for women or men. Although Burkina Faso is an exceptionally challenging environment in terms of both poverty and gender norms, it is unlikely that these characteristics alone can explain the lack of impact of SELEVER on women's empowerment.

Additionally, SELEVER did not address financial barriers, as it did not directly transfer assets to beneficiaries. In low-income settings, targeted asset transfers have previously been shown to be an essential, but not sufficient, approach for ensuring that agricultural development programs empower women and that gender-sensitive approaches can help women maintain control over these assets (van den Bold et al., 2015; Quisumbing et al., 2015; Roy et al., 2015). In other words, asset transfers can relieve start-up capital constraints, which is a primary barrier for women's production of marketable surplus (Djurfeldt 2018). In fact, most small-holder agricultural interventions in Burkina Faso include some sort of asset transfer. Helen Keller International's Enhanced Homestead Food Production program is one such model, which provided women beneficiaries with two chickens and basic agricultural implements for cultivation. Evaluations of this program found that women in study arms that received assets were able to maintain control over these assets and attained higher levels of empowerment; moreover, attitudes in the wider community become more favorable towards women's asset ownership (van den Bold et al., 2015; Olney et al., 2016) The asset transfer debate is important, as funders and implementers are often concerned about avoiding “give-away” programs. Such programs may be costly to implement, and there are often concerns that beneficiaries will become dependent on program handouts (Gautam 2019; Handa et al., 2018; Lentz et al. 2005). SELEVER was a market-based intervention that targeted relatively poor households in an exceptionally resource poor context. The program wagered much of its success on whether small-scale producers were willing to invest their own funds in infrastructure (e.g., henhouses) and veterinary care. Most households in SELEVER communities had very little to invest, especially because high poultry mortality rates can make the investment risky (Leight et al. 2022a, ). While concerns about asset transfers being too generous may be appropriate in some contexts, in the case of SELEVER, women may have been more willing to invest in poultry if they had the resources to support doing so. Labeling these assets as “women's” may have helped women maintain active control over the assets as has been demonstrated in the design of some financial tools (Buvinic and O’Donnell, 2016; O'Sullivan 2017). Overall, it may be important to reframe concerns about interventions that include asset-transfer in terms of the the relative poverty of the context or types of programs where asset transfers can make an important and meaningful difference in program outcomes.

## 6 Conclusions

We can reflect on outcomes from across the SELEVER trial using the Reach-Benefit-Empower Framework. The framework classifies gender-sensitive strategies in terms of whether they reach (e.g., include them in program activities), benefit (e.g., increase income or improve nutritional status), or empower women (increase their ability to make strategic life choices) (Johnson et al., 2018). In terms of reaching women, approximately half of households reported attending a SELEVER training in the 2019 survey (while implementation was in progress) and approximately 30% reported having attended a SELEVER training during the past year in the 2020 endline survey (Leight et al., 2022a). Evidence from the analysis of the poultry-related primary outcomes of the trial highlighted substantial positive effects of SELEVER on production of poultry owned by women (Leight et al., 2022a). However, these positive effects were counterbalanced by negative effects for poultry that was jointly owned by women and men (and weakly positive effects for poultry owned by men). This suggests some “relabeling” with households, in which exposure to the SELEVER intervention resulted in women identifying previously jointly owned poultry as their own. This finding is suggestive of small, measurable benefits to women. On the other hand, there was no impact on women's diets (Becquey et al., 2022). Moreover, in terms of empowerment, in this paper we determine that there were no measurable shifts in women's empowerment. Overall, SELEVER did an adequate job reaching women, especially for a community-based program designed to allow selective uptake; was able to generate some benefits in the area of poultry production, but not for women's diets; but there is no evidence that the program empowered women.

Considering SELEVER's lack of impact on women's empowerment and potential reasons for these outcomes, it is worth focusing on the overarching question of whether a food systems intervention can empower women. As highlighted in the emerging literature on this topic, there is evidence that gender-sensitive food systems interventions that focus on marketing have the potential to benefit women in terms of their livelihoods and potentially empower women (Johnson et al., 2018; Quisumbing et al., 2021b). In the case of SELEVER, we see some evidence of benefits to women in terms of poultry-related outcomes, but no impact on women's empowerment (Leight et al., 2022a, 2022b). Despite the increased focus on women's empowerment as a development objective, especially among rural agriculture and food systems interventions, many interventions like SELEVER fail to achieve detectable impacts on women's empowerment, despite multipronged approaches to addressing gender inequalities. The lack of impact may be attributable to limited program exposure, limited integration among the different program components, the possibility that the project did not target key areas of women's empowerment necessary for change in the context of the project, and the absence of a targeted asset transfer to bolster poultry production in a high poverty context.

## Ethical statement

Ethical review and approval was provided by the Institutional Research Board at the International Food Policy Research Institute (IRB00007490) and the Comité Éthique pour la Recherche en Santé MS/MRSI in Burkina Faso (2016-12-142);.

## Funding and acknowledgements

We acknowledge funding support for the SELEVER study from the 10.13039/100000865Bill & Melinda Gates Foundation, (Grant number: OPP1149709) and the 10.13039/501100015815CGIAR Research Program on Agriculture for Nutrition and Health (A4NH), led by IFPRI. This work was also undertaken in collaboration with the Gender, Agriculture, and Assets Project Phase Two (GAAP2). Funding for GAAP2 was provided by the 10.13039/100000865Bill & Melinda Gates Foundation (BMGF) [Grant number: OPP1125297], the 10.13039/100000200United States Agency for International Development (USAID) [Grant number: EEM-G-00-04-00013-00] and A4NH. This work would not be possible without the commitment and collaboration of the Tanager team in Ouagadougou, the study participants, or the project management efforts of Federica Argento. The opinions expressed here belong to the authors, and do not necessarily reflect those of A4NH, BMGF, CGIAR, IFPRI, or USAID.

## Role of the funding sources

Neither BMGF, USAID, nor A4NH had any role in study design; collection, analysis, or interpretation of data; writing the findings; or the decision to submit the article for publication.

## Declaration of competing interest

All authors declare that they have no conflicts of interest.

## Data Availability

The data will be made available after the conclusion of the project.

## Footnotes

1 Also known as the Women's Poultry Program to Improve Income and Nutrition.

2 The government entity responsible for livestock and fishing resources.

3 When the SELEVER baseline data were collected, pro-WEAI was still being developed, and it would not have been possible to calculate the contributions to disempowerment. SELEVER was one of the first projects to collect pro-WEAI (or earlier versions of WEAI) in Burkina Faso, and data from these projects were used in the development of the pro-WEAI indicators. Currently, however, pro-WEAI is collected more widely, and many projects will be able to draw on data collected in similar populations or use their own baseline data in conjunction with an available Stata.ado file to quickly produce statistics on the contributors to disempowerment.
