# Supplementary material for: Can a gender-sensitive integrated poultry value chain and nutrition intervention increase women's empowerment among the rural poor in Burkina Faso?
Source: J Rural Stud. 2023 May;100:103026. doi: 10.1016/j.jrurstud.2023.103026 (PMC10291270; doi:10.1016/j.jrurstud.2023.103026)
Supplement: Multimedia component 1 [file mmc1.docx]

**Appendix A: Supplementary analyses**

**Table A1. Unadjusted treatment effects on aggregate empowerment, core pro-WEAI indicators, poultry specific empowerment indicators, and indicators of health and nutrition agency**

| **Indicator** | Treatment | Standard error | n | R-squared |
| --- | --- | --- | --- | --- |
| **Aggregate empowerment indicators** |  |  |  |  |
| Empowerment score (woman) | -0.01 | 0.01 | 1,495 | 0.002 |
| Empowerment score (man) | 0.00 | 0.01 | 1,397 | 0.000 |
| Empowered (woman) | 0.00 | 0.02 | 1,495 | 0.000 |
| Empowered (man) | 0.00 | 0.04 | 1,397 | 0.000 |
| Empowerment gap (household) | 0.01 | 0.01 | 1,328 | 0.001 |
| Gender parity (household) | -0.01 | 0.03 | 1,328 | 0.000 |
| **Core pro-WEAI indicators (binary)** |  |  |  |  |
| Autonomy in income (woman) | 0.05 | 0.03 | 1,635 | 0.002 |
| Autonomy in income (man) | 0.01 | 0.03 | 1,477 | 0.000 |
| Self-efficacy (woman) | -0.01 | 0.03 | 1,635 | 0.000 |
| Self-efficacy (man) | -0.02 | 0.03 | 1,477 | 0.001 |
| Attitudes about IPV (woman) | -0.03 | 0.04 | 1,635 | 0.001 |
| Attitudes about IPV (man) | -0.04 | 0.02 | 1,477 | 0.002 |
| Respect among household members (woman) | -0.06 | 0.03 | 1,495 | 0.003 |
| Respect among household members (man) | -0.01 | 0.03 | 1,401 | 0.000 |
| Input in productive decisions (woman) | 0.00 | 0.03 | 1,635 | 0.000 |
| Input in productive decisions (man) | -0.04 | 0.01 | 1,477 | 0.007 |
| Ownership of land and other assets (woman) | -0.02 | 0.02 | 1,635 | 0.001 |
| Ownership of land and other assets (man) | -0.01 | 0.01 | 1,477 | 0.003 |
| Access to and decisions on financial services (woman) | 0.03 | 0.02 | 1,635 | 0.001 |
| Access to and decisions on financial services (man) | 0.03 | 0.04 | 1,477 | 0.001 |
| Control over use of income (woman) | -0.03 | 0.04 | 1,635 | 0.001 |
| Control over use of income (man) | 0.00 | 0.02 | 1,477 | 0.000 |
| Work balance (woman) | 0.01 | 0.04 | 1,635 | 0.000 |
| Work balance (man) | -0.03 | 0.04 | 1,472 | 0.001 |
| Visiting important locations (woman) | -0.01 | 0.03 | 1,635 | 0.000 |
| Visiting important locations (man) | -0.01 | 0.03 | 1,477 | 0.000 |
| Group membership (woman) | -0.04 | 0.03 | 1,635 | 0.002 |
| Group membership (man) | 0.06 | 0.04 | 1,477 | 0.003 |
| Membership in influential groups (woman) | -0.02 | 0.03 | 1,635 | 0.001 |
| Membership in influential groups (man) | 0.04 | 0.04 | 1,477 | 0.002 |
| **Core pro-WEAI indicators (count)** |  |  |  |  |
| New General Self-Efficacy score (woman) | -0.26 | 0.32 | 1,635 | 0.001 |
| New General Self-Efficacy score (man) | 0.10 | 0.33 | 1,477 | 0.000 |
| Number of violence situations (woman) | -0.07 | 0.12 | 1,635 | 0.000 |
| Number of violence situations (man) | -0.05 | 0.06 | 1,477 | 0.000 |
| Number of production decisions (woman) | -0.56 | 0.47 | 1,584 | 0.002 |
| Number of production decisions (man) | -0.55 | 0.43 | 1,466 | 0.002 |
| Number of assets owned (woman) | -0.05 | 0.13 | 1,635 | 0.000 |
| Number of assets owned (man) | -0.03 | 0.20 | 1,477 | 0.000 |
| Number of credit sources (woman) | 0.03 | 0.03 | 1,635 | 0.001 |
| Number of credit sources (man) | 0.09 | 0.07 | 1,477 | 0.004 |
| Number of income decisions (woman) | -0.45 | 0.42 | 1,635 | 0.001 |
| Number of income decisions (man) | -0.40 | 0.38 | 1,477 | 0.001 |
| Hours spent on work (woman) | -0.03 | 0.36 | 1,635 | 0.000 |
| Hours spent on work (man) | 0.01 | 0.44 | 1,472 | 0.000 |
| Number of visit locations (woman) | 0.06 | 0.06 | 1,637 | 0.001 |
| Number of visit locations (man) | 0.06 | 0.07 | 1,477 | 0.001 |
| Number of groups (woman) | -0.03 | 0.05 | 1,635 | 0.000 |
| Number of groups (man) | 0.11 | 0.05 | 1,477 | 0.004 |
| Number of influential groups (woman) | -0.01 | 0.04 | 1,635 | 0.000 |
| Number of influential groups (man) | 0.11 | 0.05 | 1,477 | 0.005 |
| **Poultry-specific empowerment indicators** |  |  |  |  |
| Decisions on poultry production (woman) | 0.06 | 0.24 | 1,100 | 0.000 |
| Decisions on poultry production (man) | -0.01 | 0.06 | 1,365 | 0.000 |
| Owns poultry (woman) | 0.09 | 0.05 | 1,635 | 0.009 |
| Owns poultry (man) | -0.03 | 0.02 | 1,477 | 0.003 |
| Decisions on poultry income (woman) | 0.20 | 0.25 | 1,100 | 0.002 |
| Decisions on poultry income (man) | 0.03 | 0.06 | 1,365 | 0.000 |
| Hours on poultry work (woman) | 0.00 | 0.00 | 1,635 | 0.000 |
| Hours on poultry work (man) | 0.01 | 0.03 | 1,472 | 0.000 |
| **Indicators of nutrition and health agency** |  |  |  |  |
| Decides on own health and diet (woman) | 0.00 | 0.03 | 1,635 | 0.000 |
| Decides on health and diet during pregnancy (woman) | -0.03 | 0.06 | 255 | 0.001 |
| Decides on child's diet (woman) | -0.01 | 0.03 | 1,635 | 0.000 |
| Decides on weaning and breastfeeding (woman) | 0.00 | 0.03 | 557 | 0.000 |
| Decides on seeking healthcare (woman) | 0.01 | 0.02 | 1,131 | 0.000 |
| Decides on purchasing food and health products (woman) | 0.01 | 0.03 | 1,637 | 0.000 |
| Access to food and health products (woman) | 0.02 | 0.03 | 1,637 | 0.000 |

* Statistically significant after Bonferroni correction. Bonferroni corrections were grouped based on Tables 4-8 (aggregate empowerment indicators: *p<0.0083; core pro-WEAI indicators (binary): *p<0.0042; core pro-WEAI indicators (count): *p<0.005; poultry-specific empowerment indicators: *p<0.0125; indicators of nutrition and health agency: *p<0.0071).

Standard errors are clustered at the commune level

**Table A2. Estimated treatment on the treated for aggregate empowerment, core pro-WEAI indicators, poultry specific empowerment indicators, and indicators of health and nutrition agency using propensity score weighted regression**

|  | Propensity scores using engagement in nutrition & gender | | | Propensity scores using engagement in SELEVER | | |
| --- | --- | --- | --- | --- | --- | --- |
| **Indicator** | Treatment estimate | Standard error | n | Treatment estimate | Standard error | n |
| **Aggregate empowerment indicators** |  |  |  |  |  |  |
| Empowermenft score (women) | 0.011 | 0.015 | 2857 | 0.022 | 0.023 | 2857 |
| Empowerment score (men) | 0.012 | 0.014 | 2591 | 0.016 | 0.023 | 2591 |
| Empowered (women) | 0.000 | 0.034 | 2857 | 0.098 | 0.066 | 2857 |
| Empowered (men) | 0.029 | 0.052 | 2591 | -0.042 | 0.082 | 2591 |
| Empowerment gap | 0.003 | 0.019 | 2483 | 0.003 | 0.029 | 2483 |
| Gender parity | 0.008 | 0.046 | 2483 | 0.055 | 0.080 | 2483 |
| **Core pro-WEAI indicators (binary)** |  |  |  |  |  |  |
| Autonomy in income (women) | 0.009 | 0.040 | 3246 | -0.025 | 0.073 | 3246 |
| Autonomy in income (men) | -0.077 | 0.052 | 2906 | -0.015 | 0.070 | 2906 |
| Self-efficacy (women) | -0.011 | 0.051 | 3246 | -0.057 | 0.074 | 3246 |
| Self-efficacy (men) | -0.061 | 0.042 | 2906 | -0.022 | 0.075 | 2906 |
| Attitudes about IPV (women) | -0.108 | 0.045 | 3246 | -0.047 | 0.064 | 3246 |
| Attitudes about IPV (men) | -0.089 | 0.039 | 2906 | -0.062 | 0.062 | 2906 |
| Respect among household members (women) | -0.065 | 0.046 | 3066 | -0.102 | 0.074 | 3066 |
| Respect among household members (men) | 0.025 | 0.050 | 2792 | -0.003 | 0.070 | 2792 |
| Input in productive decisions (women) | 0.061 | 0.042 | 3246 | 0.029 | 0.045 | 3246 |
| Input in productive decisions (men) | -0.028 | 0.018 | 2906 | -0.026 | 0.024 | 2906 |
| Ownership of land and other assets (women) | 0.017 | 0.034 | 3246 | 0.012 | 0.048 | 3246 |
| Ownership of land and other assets (men) | -0.001 | 0.005 | 2906 | -0.001 | 0.004 | 2906 |
| Access to and decisions on financial services (women) | 0.024 | 0.038 | 3246 | -0.015 | 0.065 | 3246 |
| Access to and decisions on financial services (men) | 0.038 | 0.043 | 2906 | 0.024 | 0.082 | 2906 |
| Control over use of income (women) | 0.044 | 0.047 | 3246 | 0.050 | 0.063 | 3246 |
| Control over use of income (men) | 0.014 | 0.029 | 2906 | 0.047 | 0.053 | 2906 |
| Work balance (women) | 0.020 | 0.046 | 3032 | 0.032 | 0.073 | 3032 |
| Work balance (men) | 0.027 | 0.051 | 2704 | -0.036 | 0.085 | 2704 |
| Visiting important locations (women) | 0.109 | 0.055 | 3246 | 0.013 | 0.072 | 3246 |
| Visiting important locations (men) | 0.062 | 0.041 | 2906 | 0.028 | 0.062 | 2906 |
| Group membership (women) | 0.056 | 0.038 | 3246 | 0.173 | 0.064 | 3246 |
| Group membership (men) | 0.140* | 0.047 | 2906 | 0.178 | 0.073 | 2906 |
| Membership in influential groups (women) | 0.048 | 0.040 | 3246 | 0.232* | 0.068 | 3246 |
| Membership in influential groups (men) | 0.090 | 0.047 | 2906 | 0.121 | 0.071 | 2906 |
| **Indicators of nutrition and health agency** |  |  |  |  |  |  |
| Decides on own health and diet (women) | -0.101 | 0.052 | 3245 | -0.192 | 0.087 | 3245 |
| Decides on health and diet during pregnancy (women) | -0.116 | 0.091 | 1001 | -0.150 | 0.150 | 1001 |
| Decides on child's diet (women) | -0.081 | 0.044 | 3245 | -0.077 | 0.080 | 3245 |
| Decides on weaning and breastfeeding (women) | -0.011 | 0.033 | 1128 | -0.033 | 0.051 | 1128 |
| Decides on seeking healthcare (women) | -0.077 | 0.032 | 2518 | -0.140 | 0.051 | 2518 |
| Decides on purchasing food and health products (women) | -0.045 | 0.026 | 3248 | 0.006 | 0.045 | 3248 |
| Access to food and health products (women) | -0.072 | 0.060 | 3248 | -0.115 | 0.060 | 3248 |
| * Statistically significant after Bonferroni correction. Bonferroni corrections were grouped based on Tables 4-8 (aggregate empowerment indicators: *p<0.0083; core pro-WEAI indicators (binary): *p<0.0042; *p<0.0125; indicators of nutrition and health agency: *p<0.0071).  Notes: Estimates shown are estimated treatment impact on the treated using difference-in-difference estimation with treated individual identified using propensity scores. The left columns show results using propensity scores based on engagement in the nutrition and gender components of SELEVER; the right columns show results using propensity scores based on engagement in the full SELEVER intervention. Standard errors are clustered at the commune level. N indicates total number of observations in the difference-in-differences estimation. | | | | | | |

**Table A3. Estimated program impacts adjusting for poultry flock size at baseline**

| **Indicator** | Treatment | Standard error | Large producer (>=20 mature birds at baseline) | Standard error | Interaction term (treatment X large producer) | Standard error | n |
| --- | --- | --- | --- | --- | --- | --- | --- |
| **Aggregate empowerment indicators** |  |  |  |  |  |  |  |
| Empowerment score (women) | -0.007 | 0.016 | -0.013 | 0.015 | -0.009 | 0.020 | 1492 |
| Empowerment score (men) | -0.002 | 0.016 | -0.008 | 0.013 | 0.002 | 0.018 | 1398 |
| Empowered (women) | 0.022 | 0.025 | 0.024 | 0.021 | -0.044 | 0.033 | 1492 |
| Empowered (men) | -0.028 | 0.044 | -0.030 | 0.036 | 0.056 | 0.053 | 1398 |
| Empowerment gap | 0.011 | 0.017 | 0.005 | 0.018 | 0.000 | 0.026 | 1328 |
| Gender parity | 0.019 | 0.035 | 0.012 | 0.038 | -0.046 | 0.055 | 1328 |
| **Core pro-WEAI indicators (binary)** |  |  |  |  |  |  |  |
| Autonomy in income (women) | 0.039 | 0.041 | -0.024 | 0.031 | 0.024 | 0.047 | 1630 |
| Autonomy in income (men) | 0.009 | 0.036 | -0.049 | 0.034 | 0.013 | 0.048 | 1474 |
| Self-efficacy (women) | -0.007 | 0.042 | -0.025 | 0.041 | 0.000 | 0.057 | 1630 |
| Self-efficacy (men) | -0.029 | 0.033 | -0.002 | 0.032 | -0.004 | 0.042 | 1474 |
| Attitudes about IPV (women) | -0.026 | 0.048 | -0.044 | 0.038 | -0.008 | 0.054 | 1630 |
| Attitudes about IPV (men) | -0.025 | 0.035 | 0.009 | 0.036 | -0.026 | 0.052 | 1474 |
| Respect among household members (women) | -0.039 | 0.030 | 0.014 | 0.038 | -0.027 | 0.049 | 1492 |
| Respect among household members (men) | -0.017 | 0.035 | -0.023 | 0.034 | 0.018 | 0.047 | 1402 |
| Input in productive decisions (women) | -0.005 | 0.037 | -0.046 | 0.033 | 0.027 | 0.045 | 1630 |
| Input in productive decisions (men) | -0.029 | 0.018 | -0.012 | 0.014 | -0.010 | 0.020 | 1474 |
| Ownership of land and other assets (women) | -0.012 | 0.023 | 0.020 | 0.024 | -0.003 | 0.033 | 1630 |
| Ownership of land and other assets (men) | -0.008 | 0.007 | 0.003 | 0.003 | 0.000 | 0.008 | 1474 |
| Access to and decisions on financial services (women) | 0.053 | 0.031 | 0.007 | 0.030 | -0.055 | 0.042 | 1630 |
| Access to and decisions on financial services (men) | 0.024 | 0.048 | -0.005 | 0.034 | 0.014 | 0.043 | 1474 |
| Control over use of income (women) | -0.028 | 0.049 | -0.019 | 0.041 | -0.001 | 0.060 | 1630 |
| Control over use of income (men) | 0.005 | 0.029 | -0.048 | 0.029 | -0.003 | 0.036 | 1474 |
| Work balance (women) | 0.039 | 0.038 | 0.017 | 0.035 | -0.058 | 0.042 | 1630 |
| Work balance (men) | -0.022 | 0.047 | 0.003 | 0.039 | -0.009 | 0.056 | 1469 |
| Visiting important locations (women) | 0.042 | 0.035 | 0.055 | 0.040 | -0.118 | 0.051 | 1630 |
| Visiting important locations (men) | 0.026 | 0.035 | 0.056 | 0.047 | -0.096 | 0.059 | 1474 |
| Group membership (women) | -0.064 | 0.043 | -0.042 | 0.038 | 0.061 | 0.052 | 1630 |
| Group membership (men) | 0.019 | 0.042 | -0.012 | 0.032 | 0.071 | 0.048 | 1474 |
| Membership in influential groups (women) | -0.050 | 0.040 | -0.051 | 0.039 | 0.067 | 0.055 | 1630 |
| Membership in influential groups (men) | 0.010 | 0.044 | -0.018 | 0.036 | 0.069 | 0.051 | 1474 |
| **Indicators of nutrition and health agency** |  |  |  |  |  |  |  |
| Decides on own health and diet (women) | 0.048 | 0.039 | 0.026 | 0.034 | -0.109 | 0.054 | 1630 |
| Decides on health and diet during pregnancy (women) | -0.033 | 0.074 | 0.004 | 0.074 | 0.006 | 0.108 | 256 |
| Decides on child's diet (women) | 0.018 | 0.034 | 0.051 | 0.032 | -0.064 | 0.047 | 1630 |
| Decides on weaning and breastfeeding (women) | -0.017 | 0.032 | 0.013 | 0.031 | 0.025 | 0.045 | 556 |
| Decides on seeking healthcare (women) | 0.025 | 0.022 | 0.013 | 0.022 | -0.027 | 0.028 | 1128 |
| Decides on purchasing food and health products (women) | 0.003 | 0.030 | -0.012 | 0.028 | 0.024 | 0.039 | 1632 |
| Access to food and health products (women) | 0.016 | 0.041 | -0.039 | 0.034 | 0.012 | 0.046 | 1632 |
| * Statistically significant after Bonferroni correction. Bonferroni corrections were grouped based on Tables 4-8 (aggregate empowerment indicators: *p<0.0083; core pro-WEAI indicators (binary): *p<0.0042; *p<0.0125; indicators of nutrition and health agency: *p<0.0071). | | | | | | | |
| Notes: Estimates shown are treatment effect estimates using analysis of covariance models, controlling for whether the household was a large poultry producer (20 birds or more) and the interaction between treatment and large producer. Models also control for household size and age of respondent. Standard errors are clustered at the commune level. | | | | | | | |

**Table A4. Treatment effect estimates, second level randomization (Control vs. SELEVER vs. SELEVER+WASH)**

| **Indicator** | SELEVER  (vs control) | Standard error | SELEVER+WASH  (vs control) | Standard error | n | R-squared | Post-test p-value  (SELEVER vs SELEVER+WASH) |
| --- | --- | --- | --- | --- | --- | --- | --- |
| **Aggregate empowerment indicators** |  |  |  |  |  |  |  |
| Empowerment score (woman) | 0.00 | 0.02 | -0.02 | 0.01 | 1,106 | 0.017 | 0.289 |
| Empowerment score (man) | 0.01 | 0.02 | 0.01 | 0.02 | 1,036 | 0.006 | 0.987 |
| Empowered (woman) | 0.03 | 0.03 | -0.02 | 0.03 | 1,106 | 0.007 | 0.136 |
| Empowered (man) | 0.03 | 0.05 | 0.04 | 0.05 | 1,036 | 0.006 | 0.810 |
| Empowerment gap (household) | 0.01 | 0.02 | 0.04 | 0.02 | 981 | 0.021 | 0.128 |
| Gender parity (household) | 0.02 | 0.04 | -0.06 | 0.03 | 981 | 0.013 | 0.052 |
| **Core pro-WEAI indicators (binary)** |  |  |  |  |  |  |  |
| Autonomy in income (woman) | 0.02 | 0.04 | 0.06 | 0.04 | 1,213 | 0.003 | 0.343 |
| Autonomy in income (man) | -0.02 | 0.04 | -0.04 | 0.03 | 1,089 | 0.002 | 0.396 |
| Self-efficacy (woman) | 0.02 | 0.04 | 0.06 | 0.04 | 1,213 | 0.007 | 0.402 |
| Self-efficacy (man) | -0.01 | 0.04 | -0.03 | 0.04 | 1,089 | 0.065 | 0.736 |
| Attitudes about IPV (woman) | -0.06 | 0.06 | -0.05 | 0.05 | 1,213 | 0.009 | 0.788 |
| Attitudes about IPV (man) | -0.05 | 0.03 | -0.04 | 0.03 | 1,089 | 0.005 | 0.670 |
| Respect among household members (woman) | -0.03 | 0.04 | -0.04 | 0.04 | 1,106 | 0.004 | 0.909 |
| Respect among household members (man) | -0.02 | 0.04 | 0.00 | 0.04 | 1,039 | 0.002 | 0.551 |
| Input in productive decisions (woman) | 0.01 | 0.03 | -0.03 | 0.04 | 1,213 | 0.009 | 0.321 |
| Input in productive decisions (man) | -0.01 | 0.02 | -0.03 | 0.02 | 1,089 | 0.003 | 0.560 |
| Ownership of land and other assets (woman) | 0.01 | 0.02 | 0.00 | 0.02 | 1,213 | 0.001 | 0.780 |
| Ownership of land and other assets (man) | -0.01 | 0.01 | -0.01 | 0.01 | 1,089 | 0.007 | 0.640 |
| Access to and decisions on financial services (woman) | 0.03 | 0.04 | 0.00 | 0.03 | 1,213 | 0.005 | 0.269 |
| Access to and decisions on financial services (man) | 0.06 | 0.06 | 0.10 | 0.07 | 1,089 | 0.014 | 0.477 |
| Control over use of income (woman) | -0.08 | 0.05 | -0.09 | 0.04 | 1,213 | 0.014 | 0.710 |
| Control over use of income (man) | 0.01 | 0.03 | -0.03 | 0.03 | 1,089 | 0.009 | 0.236 |
| Work balance (woman) | -0.06 | 0.05 | -0.08 | 0.05 | 1,213 | 0.035 | 0.528 |
| Work balance (man) | -0.05 | 0.05 | -0.07 | 0.05 | 1,086 | 0.032 | 0.625 |
| Visiting important locations (woman) | 0.06 | 0.04 | 0.01 | 0.04 | 1,213 | 0.004 | 0.139 |
| Visiting important locations (man) | 0.03 | 0.04 | 0.04 | 0.04 | 1,089 | 0.012 | 0.908 |
| Group membership (woman) | 0.01 | 0.04 | -0.05 | 0.04 | 1,213 | 0.009 | 0.213 |
| Group membership (man) | 0.08 | 0.05 | 0.11 | 0.05 | 1,089 | 0.015 | 0.531 |
| Membership in influential groups (woman) | 0.05 | 0.04 | -0.01 | 0.03 | 1,213 | 0.011 | 0.167 |
| Membership in influential groups (man) | 0.10 | 0.05 | 0.08 | 0.06 | 1,089 | 0.014 | 0.746 |
| **Core pro-WEAI indicators (count)** |  |  |  |  |  |  |  |
| New General Self-Efficacy score (woman) | -0.47 | 0.42 | -0.12 | 0.42 | 1,213 | 0.010 | 0.492 |
| New General Self-Efficacy score (man) | -0.04 | 0.39 | -0.04 | 0.39 | 1,089 | 0.104 | 0.996 |
| Number of violence situations (woman) | -0.18 | 0.20 | -0.04 | 0.16 | 1,213 | 0.007 | 0.424 |
| Number of violence situations (man) | -0.04 | 0.08 | 0.00 | 0.09 | 1,089 | 0.003 | 0.651 |
| Number of production decisions (woman) | -0.04 | 0.68 | -0.03 | 0.62 | 1,176 | 0.003 | 0.988 |
| Number of production decisions (man) | -1.07 | 0.44 | 0.52 | 0.53 | 1,080 | 0.022 | 0.007 |
| Number of assets owned (woman) | 0.09 | 0.20 | -0.02 | 0.16 | 1,213 | 0.007 | 0.575 |
| Number of assets owned (man) | 0.20 | 0.25 | 0.29 | 0.25 | 1,089 | 0.009 | 0.749 |
| Number of credit sources (woman) | 0.04 | 0.04 | -0.01 | 0.04 | 1,213 | 0.004 | 0.223 |
| Number of credit sources (man) | 0.16 | 0.09 | 0.15 | 0.09 | 1,089 | 0.016 | 0.901 |
| Number of income decisions (woman) | -0.34 | 0.61 | -0.09 | 0.59 | 1,213 | 0.006 | 0.693 |
| Number of income decisions (man) | -1.04 | 0.40 | 0.57 | 0.50 | 1,089 | 0.024 | 0.003* |
| Hours spent on work (woman) | 0.49 | 0.51 | 0.84 | 0.53 | 1,213 | 0.024 | 0.424 |
| Hours spent on work (man) | 0.57 | 0.65 | 0.79 | 0.64 | 1,086 | 0.049 | 0.712 |
| Number of visit locations (woman) | 0.11 | 0.09 | 0.08 | 0.09 | 1,215 | 0.004 | 0.703 |
| Number of visit locations (man) | 0.16 | 0.10 | 0.17 | 0.10 | 1,089 | 0.027 | 0.944 |
| Number of groups (woman) | 0.01 | 0.06 | -0.06 | 0.06 | 1,213 | 0.009 | 0.317 |
| Number of groups (man) | 0.08 | 0.08 | 0.17 | 0.08 | 1,089 | 0.011 | 0.261 |
| Number of influential groups (woman) | 0.07 | 0.06 | 0.00 | 0.05 | 1,213 | 0.010 | 0.281 |
| Number of influential groups (man) | 0.14 | 0.07 | 0.18 | 0.08 | 1,089 | 0.012 | 0.691 |
| **Poultry-specific empowerment indicators** |  |  |  |  |  |  |  |
| Decisions on poultry production (woman) | 0.46 | 0.29 | 0.30 | 0.32 | 808 | 0.022 | 0.662 |
| Decisions on poultry production (man) | 0.01 | 0.06 | 0.13 | 0.09 | 1,005 | 0.007 | 0.187 |
| Owns poultry (woman) | 0.13 | 0.06 | 0.13 | 0.05 | 1,213 | 0.029 | 0.980 |
| Owns poultry (man) | -0.06 | 0.03 | -0.01 | 0.02 | 1,089 | 0.021 | 0.123 |
| Decisions on poultry income (woman) | 0.42 | 0.28 | 0.36 | 0.32 | 808 | 0.017 | 0.859 |
| Decisions on poultry income (man) | 0.02 | 0.06 | 0.15 | 0.08 | 1,005 | 0.009 | 0.110 |
| Hours on poultry work (woman) | 0.00 | 0.00 | 0.00 | 0.01 | 1,213 | 0.003 | 0.287 |
| Hours on poultry work (man) | -0.06 | 0.05 | -0.03 | 0.05 | 1,086 | 0.008 | 0.300 |
| **Indicators of nutrition and health agency** |  |  |  |  |  |  |  |
| Decides on own health and diet (woman) | 0.01 | 0.03 | 0.04 | 0.03 | 1,213 | 0.002 | 0.257 |
| Decides on health and diet during pregnancy (woman) | 0.08 | 0.07 | -0.14 | 0.07 | 190 | 0.055 | 0.011* |
| Decides on child's diet (woman) | 0.02 | 0.04 | 0.02 | 0.04 | 1,213 | 0.001 | 0.890 |
| Decides on weaning and breastfeeding (woman) | -0.02 | 0.04 | 0.01 | 0.04 | 405 | 0.004 | 0.329 |
| Decides on seeking healthcare (woman) | 0.04 | 0.03 | 0.00 | 0.02 | 837 | 0.019 | 0.251 |
| Decides on purchasing food and health products (woman) | 0.04 | 0.03 | 0.03 | 0.04 | 1,215 | 0.007 | 0.761 |
| Access to food and health products (woman) | 0.05 | 0.04 | 0.06 | 0.05 | 1,215 | 0.005 | 0.886 |

*Statistically significant after Bonferroni correction. Bonferroni corrections were grouped based on Tables 4-8 (aggregate empowerment indicators: *p<0.0083; core pro-WEAI indicators (binary): *p<0.0042; core pro-WEAI indicators (count): *p<0.005; poultry-specific empowerment indicators: *p<0.0125; indicators of nutrition and health agency: *p<0.0071).

Standard errors are clustered at the commune level.

**Table A5. Treatment effect estimates, adjusted for pre-post COVID lockdown**

| **Indicator** | Treatment | Standard error | Post-COVID | Standard error | Interaction term (treatment X post-COVID) | Standard error | n | R-squared |
| --- | --- | --- | --- | --- | --- | --- | --- | --- |
| **Aggregate empowerment indicators** |  |  |  |  |  |  |  |  |
| Empowerment score (woman) | -0.03 | 0.02 | -0.02 | 0.02 | 0.02 | 0.03 | 1,492 | 0.013 |
| Empowerment score (man) | 0.02 | 0.03 | 0.04 | 0.02 | -0.02 | 0.03 | 1,398 | 0.012 |
| Empowered (woman) | -0.09 | 0.04 | -0.08* | 0.03 | 0.11 | 0.04 | 1,492 | 0.007 |
| Empowered (man) | 0.00 | 0.07 | 0.07 | 0.06 | 0.00 | 0.08 | 1,398 | 0.009 |
| Empowerment gap (household) | 0.04 | 0.03 | 0.06* | 0.02 | -0.04 | 0.03 | 1,328 | 0.017 |
| Gender parity (household) | -0.03 | 0.05 | -0.05 | 0.04 | 0.04 | 0.06 | 1,328 | 0.008 |
| **Core pro-WEAI indicators (binary)** |  |  |  |  |  |  |  |  |
| Autonomy in income (woman) | 0.02 | 0.06 | -0.04 | 0.04 | 0.03 | 0.07 | 1,630 | 0.003 |
| Autonomy in income (man) | -0.03 | 0.06 | -0.06 | 0.05 | 0.05 | 0.06 | 1,474 | 0.001 |
| Self-efficacy (woman) | 0.05 | 0.06 | 0.09 | 0.05 | -0.07 | 0.07 | 1,630 | 0.009 |
| Self-efficacy (man) | -0.02 | 0.06 | 0.05 | 0.05 | -0.01 | 0.07 | 1,474 | 0.068 |
| Attitudes about IPV (woman) | -0.11 | 0.09 | -0.06 | 0.08 | 0.09 | 0.10 | 1,630 | 0.008 |
| Attitudes about IPV (man) | -0.11 | 0.04 | -0.05 | 0.04 | 0.09 | 0.05 | 1,474 | 0.004 |
| Respect among household members (woman) | -0.10 | 0.06 | -0.05 | 0.05 | 0.05 | 0.07 | 1,492 | 0.009 |
| Respect among household members (man) | 0.01 | 0.05 | -0.01 | 0.04 | -0.03 | 0.06 | 1,402 | 0.003 |
| Input in productive decisions (woman) | -0.03 | 0.06 | -0.02 | 0.05 | 0.05 | 0.07 | 1,630 | 0.007 |
| Input in productive decisions (man) | -0.05 | 0.03 | 0.00 | 0.03 | 0.02 | 0.03 | 1,474 | 0.009 |
| Ownership of land and other assets (woman) | 0.01 | 0.05 | 0.06 | 0.03 | -0.02 | 0.05 | 1,630 | 0.004 |
| Ownership of land and other assets (man) | -0.02 | 0.02 | 0.01 | 0.01 | 0.02 | 0.02 | 1,474 | 0.017 |
| Access to and decisions on financial services (woman) | 0.08 | 0.06 | 0.03 | 0.05 | -0.06 | 0.06 | 1,630 | 0.005 |
| Access to and decisions on financial services (man) | 0.14* | 0.04 | 0.26* | 0.04 | -0.12 | 0.06 | 1,474 | 0.037 |
| Control over use of income (woman) | -0.09 | 0.06 | 0.00 | 0.06 | 0.08 | 0.08 | 1,630 | 0.010 |
| Control over use of income (man) | 0.04 | 0.03 | 0.02 | 0.02 | -0.05 | 0.04 | 1,474 | 0.007 |
| Work balance (woman) | -0.13 | 0.05 | -0.32* | 0.04 | 0.16 | 0.06 | 1,630 | 0.073 |
| Work balance (man) | -0.09 | 0.05 | -0.18* | 0.04 | 0.06 | 0.06 | 1,469 | 0.040 |
| Visiting important locations (woman) | -0.01 | 0.06 | -0.05 | 0.05 | -0.01 | 0.06 | 1,630 | 0.003 |
| Visiting important locations (man) | 0.01 | 0.05 | -0.02 | 0.04 | -0.04 | 0.06 | 1,474 | 0.009 |
| Group membership (woman) | -0.06 | 0.07 | 0.03 | 0.05 | 0.03 | 0.07 | 1,630 | 0.008 |
| Group membership (man) | 0.08 | 0.08 | 0.17 | 0.07 | -0.01 | 0.09 | 1,474 | 0.024 |
| Membership in influential groups (woman) | 0.00 | 0.05 | 0.11 | 0.04 | -0.02 | 0.06 | 1,630 | 0.013 |
| Membership in influential groups (man) | 0.09 | 0.07 | 0.19 | 0.07 | -0.04 | 0.08 | 1,474 | 0.024 |
| **Core pro-WEAI indicators (count)** |  |  |  |  |  |  |  |  |
| New General Self-Efficacy score (woman) | 0.43 | 0.67 | 0.32 | 0.47 | -0.87 | 0.76 | 1,630 | 0.013 |
| New General Self-Efficacy score (man) | 0.51 | 0.65 | 0.41 | 0.64 | -0.60 | 0.72 | 1,474 | 0.092 |
| Number of violence situations (woman) | -0.39 | 0.23 | -0.19 | 0.21 | 0.40 | 0.27 | 1,630 | 0.008 |
| Number of violence situations (man) | -0.10 | 0.10 | -0.10 | 0.08 | 0.06 | 0.12 | 1,474 | 0.002 |
| Number of production decisions (woman) | -1.04 | 0.83 | 1.03 | 0.80 | 0.73 | 0.99 | 1,580 | 0.015 |
| Number of production decisions (man) | -0.49 | 0.72 | 0.04 | 0.50 | -0.10 | 0.88 | 1,464 | 0.025 |
| Number of assets owned (woman) | 0.14 | 0.28 | 0.51 | 0.25 | -0.18 | 0.32 | 1,630 | 0.014 |
| Number of assets owned (man) | 0.10 | 0.25 | 0.86* | 0.19 | -0.06 | 0.34 | 1,474 | 0.031 |
| Number of credit sources (woman) | 0.09 | 0.07 | 0.04 | 0.06 | -0.07 | 0.07 | 1,630 | 0.004 |
| Number of credit sources (man) | 0.19* | 0.04 | 0.34* | 0.05 | -0.10 | 0.09 | 1,474 | 0.037 |
| Number of income decisions (woman) | -1.11 | 0.85 | 0.58 | 0.81 | 0.95 | 0.98 | 1,630 | 0.013 |
| Number of income decisions (man) | -0.07 | 0.60 | -0.15 | 0.42 | -0.42 | 0.74 | 1,474 | 0.025 |
| Hours spent on work (woman) | 1.30* | 0.32 | 3.39* | 0.26 | -1.43* | 0.41 | 1,630 | 0.090 |
| Hours spent on work (man) | 0.91 | 0.66 | 3.68* | 0.51 | -0.82 | 0.71 | 1,469 | 0.127 |
| Number of visit locations (woman) | -0.04 | 0.10 | -0.27* | 0.08 | 0.10 | 0.12 | 1,632 | 0.009 |
| Number of visit locations (man) | 0.22 | 0.15 | 0.04 | 0.14 | -0.23 | 0.17 | 1,474 | 0.014 |
| Number of groups (woman) | -0.11 | 0.09 | -0.02 | 0.08 | 0.11 | 0.11 | 1,630 | 0.008 |
| Number of groups (man) | 0.02 | 0.16 | 0.01 | 0.15 | 0.11 | 0.17 | 1,474 | 0.010 |
| Number of influential groups (woman) | -0.01 | 0.08 | 0.10 | 0.07 | 0.02 | 0.09 | 1,630 | 0.010 |
| Number of influential groups (man) | 0.11 | 0.12 | 0.11 | 0.11 | 0.02 | 0.13 | 1,474 | 0.011 |
| **Poultry-specific empowerment indicators** |  |  |  |  |  |  |  |  |
| Decisions on poultry production (woman) | -0.08 | 0.55 | 0.77 | 0.36 | 0.30 | 0.60 | 1,098 | 0.038 |
| Decisions on poultry production (man) | 0.04 | 0.09 | 0.07 | 0.08 | -0.07 | 0.11 | 1,364 | 0.002 |
| Owns poultry (woman) | 0.13 | 0.10 | 0.12 | 0.07 | -0.04 | 0.11 | 1,630 | 0.024 |
| Owns poultry (man) | -0.03 | 0.04 | -0.02 | 0.03 | 0.01 | 0.05 | 1,474 | 0.015 |
| Decisions on poultry income (woman) | -0.09 | 0.54 | 0.61 | 0.38 | 0.48 | 0.60 | 1,098 | 0.034 |
| Decisions on poultry income (man) | 0.22 | 0.10 | 0.10 | 0.08 | -0.24 | 0.12 | 1,364 | 0.005 |
| Hours on poultry work (woman) | 0.01 | 0.00 | 0.01* | 0.00 | -0.01 | 0.00 | 1,630 | 0.002 |
| Hours on poultry work (man) | -0.19 | 0.10 | -0.24 | 0.10 | 0.24 | 0.11 | 1,469 | 0.024 |
| **Indicators of nutrition and health agency** |  |  |  |  |  |  |  |  |
| Decides on own health and diet (woman) | -0.07 | 0.04 | 0.00 | 0.04 | 0.08 | 0.05 | 1,630 | 0.003 |
| Decides on health and diet during pregnancy (woman) | 0.01 | 0.13 | 0.04 | 0.08 | -0.06 | 0.14 | 256 | 0.004 |
| Decides on child's diet (woman) | 0.00 | 0.04 | 0.09 | 0.04 | 0.00 | 0.06 | 1,630 | 0.006 |
| Decides on weaning and breastfeeding (woman) | 0.05 | 0.05 | 0.02 | 0.04 | -0.07 | 0.06 | 556 | 0.003 |
| Decides on seeking healthcare (woman) | 0.06 | 0.04 | 0.05 | 0.03 | -0.06 | 0.04 | 1,128 | 0.016 |
| Decides on purchasing food and health products (woman) | 0.04 | 0.05 | 0.03 | 0.04 | -0.03 | 0.06 | 1,632 | 0.005 |
| Access to food and health products (woman) | 0.09 | 0.06 | 0.10 | 0.05 | -0.09 | 0.07 | 1,632 | 0.006 |

* Statistically significant after Bonferroni correction. Bonferroni corrections were grouped based on Tables 4-8 (aggregate empowerment indicators: *p<0.0083; core pro-WEAI indicators (binary): *p<0.0042; core pro-WEAI indicators (count): *p<0.005; poultry-specific empowerment indicators: *p<0.0125; indicators of nutrition and health agency: *p<0.0071).

Notes: Estimates shown are treatment effect estimates using analysis of covariance models, controlling for whether the endline interview was conducted after the beginning of the COVID-19 pandemic and the interaction between treatment and post-COVID interviews. “Post-COVID” is a binary variable where 1 indicates individuals whose endline interviews were conducted before the beginning of the COVID-19 pandemic (January to March 2020) and 0 indicates individuals whose endline interviews were conducted after the beginning of the COVID-19 pandemic (June to August 2020). For the household-level variables (empowerment gap and gender parity), households were considered post-COVID if either the man or woman was interviewed after the beginning of the pandemic. Models also control for household size and age of respondent. Standard errors are clustered at the commune level.
